# LCM and RNA-seq analyses revealed roles of cell cycle and translational regulation and homoeolog expression bias in cotton fiber cell initiation

**DOI:** 10.1186/s12864-021-07579-1

**Published:** 2021-04-29

**Authors:** Atsumi Ando, Ryan C. Kirkbride, Don C. Jones, Jane Grimwood, Z. Jeffrey Chen

**Affiliations:** 1 Department of Molecular Biosciences, and Center for Computational Biology and Bioinformatics, The University of Texas at Austin, Austin, TX 78712 USA; 2 Agriculture and Environmental Research, Cotton Incorporated, Cary, NC USA; 3 HudsonAlpha Institute for Biotechnology, Huntsville, AL USA

## Abstract

**Background:**

Cotton fibers provide a powerful model for studying cell differentiation and elongation. Each cotton fiber is a singular and elongated cell derived from epidermal-layer cells of a cotton seed. Efforts to understand this dramatic developmental shift have been impeded by the difficulty of separation between fiber and epidermal cells.

**Results:**

Here we employed laser-capture microdissection (LCM) to separate these cell types. RNA-seq analysis revealed transitional differences between fiber and epidermal-layer cells at 0 or 2 days post anthesis. Specifically, down-regulation of putative cell cycle genes was coupled with upregulation of ribosome biosynthesis and translation-related genes, which may suggest their respective roles in fiber cell initiation. Indeed, the amount of fibers in cultured ovules was increased by cell cycle progression inhibitor, Roscovitine, and decreased by ribosome biosynthesis inhibitor, Rbin-1. Moreover, subfunctionalization of homoeologs was pervasive in fiber and epidermal cells, with expression bias towards 10% more D than A homoeologs of cell cycle related genes and 40–50% more D than A homoeologs of ribosomal protein subunit genes. Key cell cycle regulators were predicted to be epialleles in allotetraploid cotton. MYB-transcription factor genes displayed expression divergence between fibers and ovules. Notably, many phytohormone-related genes were upregulated in ovules and down-regulated in fibers, suggesting spatial-temporal effects on fiber cell development.

**Conclusions:**

Fiber cell initiation is accompanied by cell cycle arrest coupled with active ribosome biosynthesis, spatial-temporal regulation of phytohormones and MYB transcription factors, and homoeolog expression bias of cell cycle and ribosome biosynthesis genes. These valuable genomic resources and molecular insights will help develop breeding and biotechnological tools to improve cotton fiber production.

**Supplementary Information:**

The online version contains supplementary material available at 10.1186/s12864-021-07579-1.

## Background

Cotton is the largest renewable source of textile fiber in the world and is an important oil crop [1, 2]. Most widely cultivated cottons are allotetraploid, consisting of two sets of chromosomes from different origins across the old and new world, with the “mother” (A-genome like species) from Africa and the “father” (D-genome like species) from the Americas [3]. The polyploidization took place in the new world ~ 1.5 million years ago [4], and resulted in five extant species that diversified over 300,000–600,000 years [4, 5]. Over the last 8000 years, two of them, Upland (*Gossypium hirsutum* L*.*) and Pima (*G. barbadense* L*.*) cottons were independently domesticated in NW S. America and the Yucatan Peninsula of Mexico, respectively, under strong human selection, leading to the modern annualized crops [6]. To date, Upland cotton dominates in ~ 94% of world cotton production, with the remainder (~ 5%) being Pima cotton for its superior fiber quality [1], and other diploid cottons adapted to certain growth environments [7].

Cotton fiber is seed hair, and each individual cotton fiber initiates from a single epidermal-like cell from the ovular epidermis, which elongates dramatically to reach up to ~ 6 cm in length, one of the longest singular cells in the plant kingdom [2]. Each cotton seed has over 20,000 semi-synchronically developed fiber cells, representing 20–30% of cells in the epidermal layer of the seed [8, 9]. There are two types of fiber, lint and fuzz fibers [10, 11], and only the lint fibers are ginned and used in textiles. Lint fiber cells are initiated before or on the day of anthesis (0 Day Post Anthesis, DPA), which induces production and transportation of the phytohormones auxin and gibberellin to promote fiber cell elongation [12]. Fuzz fiber is produced approximately 5 days after the development of lint fibers and is much shorter than the lint fiber. Fiber cells undergo overlapping stages of elongation, cellulose biosynthesis, and maturation [13]. During elongation phase, primary cell wall is synthesized, and the cell extends through some combination of anisotropic expansion and tip growth [11]. Once fibers have reached full length, they transition to the stage of cellulose and secondary cell wall biosynthesis, which is accompanied by the maturation phase where fibers, now comprising ~ 95% pure cellulose, become metabolically inactive, leading to cell death, which happens before the boll opens [11].

In developing fiber cells, cell cycle is arrested at G1 or S phase [14]. Cell differentiation and cell cycle regulation are linked in plants [15], which involves cyclin-dependent kinases (CDKs) [16], phytohormones (auxin and gibberellins), and key transcription factors. Cell cycle exit without mitotic division often leads to endoreduplication, which is common in eudicot plants [17]. Previous studies showed that DNA content was increased by endoreplication during early fiber development [18, 19], which is accompanied with small RNA and DNA methylation changes in early fiber cells [20–22]. In human cancer cells, tumor suppressor p53 has been shown to induce cell-cycle arrest in response to impaired ribosome biogenesis [23], and vice versa, cell cycle arrest during tumor metastasis is fueled by upregulation of ribosome biogenesis [24].

Phytohormones and transcription factors also play crucial roles in fiber cell development [2, 25–27]. Auxin, brassinosteroids, ethylene, and gibberellins promote or induce fiber cell development [28–30], whereas cytokinin and abscisic acid inhibit fiber cell development [31]. Previous studies have shown that auxin accumulates in ovules before fertilization, peaking around 2–3 DPA, with the level gradually decreasing through the elongation stage [12], while abscisic acid gradually accumulates in fiber cells during the initiation and elongation stages, with a peak around 10 DPA, followed by a decrease in later stages [32].

MYB (myeloblastosis) transcription factors are required for development of leaf trichomes and seed hair or fiber cells [33–36]. Several MYB transcription factor families are shown to regulate fiber cell initiation and elongation [34–37], while some MYB transcription factor members do not have an obvious role in cotton fiber cell development [38].

Regulatory networks involving phytohormones and transcription factors during early stages of fiber cell development remain poorly understood, partly because it is technically difficult to isolate fiber and epidermal cells during fiber cell initiation. Here we performed RNA-seq analysis using the fiber and epidermal cells that were separated by Laser Capture Microdissection (LCM) in early stages (0 and 2 DPA). We compared transcriptomes of LCM samples between the wild-type and fiber-less mutant (both in Upland background) and between Upland and Pima cottons, which revealed expression differences in the genes associated with cell cycle regulation and ribosome biosynthesis. Using pharmacological inhibitors for these pathways, we showed that cell cycle regulation and ribosome biogenesis affect fiber cell initiation and elongation in the ovules cultured in vitro. Moreover, phytohormone related genes were upregulated in the ovules and down-regulated in early fibers, suggesting a spatial-temporal role in fiber cell development. MYB-transcription factor related genes displayed expression divergence between fibers and ovules, while key cell cycle regulators were predicted to be epialleles [39]. Together, these results support a new role for cell cycle and translational regulation in fiber cell initiation and elongation, which is accompanied by spatial-temporal regulation of transcriptional and phytohormonal networks.

## Results

### Fiber cell initiation in GhTM-1, Gb3–79, and GhMD17

**Fig. 1:**
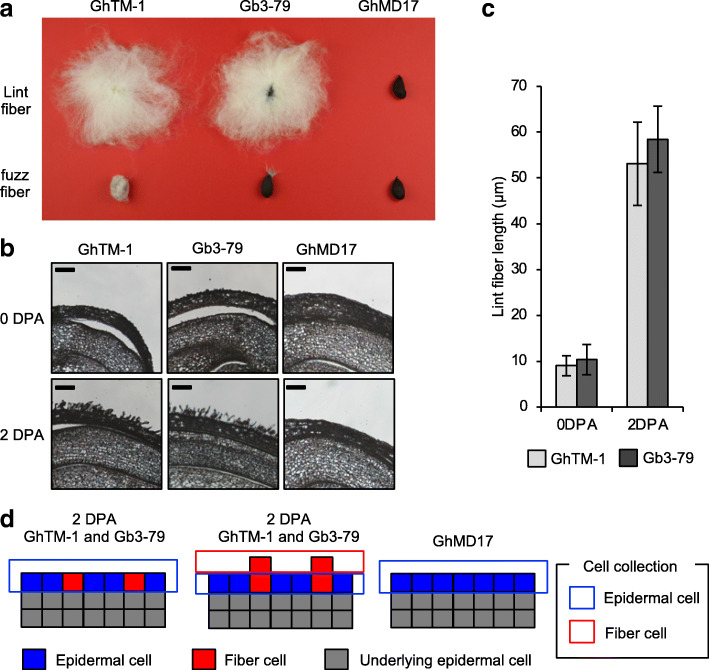
Seed and fiber morphology in GhTM-1, Gb3–79, and GhMD17. **a** Fiber morphologies on mature cotton seeds before (top) and after (bottom) lint fiber removal show both lint and fuzz fibers in *G. hirsutum* TM-1 (GhTM-1), lint finer in *G. barbadense* 3–79 (Gb3–79), and no fiber in GhMD17. **b** Paraffin embedded tissue of the ovule at 0 or 2 day post anthesis (DPA) in GhTM-1, Gb3–79, and GhMD17. Scale bars = 100 μm. **c** Lint fiber length (μm) in the ovules at 0 or 2 DPA in GhTM-1 (light gray) and Gb3–79 (black). Fiber length in a given stage was similar between GhTM-1 and Gb3–79 (Student *t*-test). **d** Diagram of epidermis (outer integument) for tissue collection using laser-capture microdissection (LCM) from the ovules at 0 or 2 DPA in GhTM-1, Gb3–79, and GhMD17. Colors indicate epidermal cell (blue), fiber cell (red), and underlying epidermal cell (gray). Dissected regions of epidermal cells (blue) and fiber cells (red) are indicated by rectangle boxes

Our study involved two cultivated cottons, *G. hirsutum* L. acc. TM-1 (GhTM-1) and *G. barbadense* L. acc. 3–79 (Gb3–79), and the fiber-less mutant GhMD17 (*G. hirsutum* background). MD17 is a naturally occurring mutant with naked seed (lintless and fuzz fiberless) phenotype, which is proposed to be controlled by three genetic loci *N*_*1*_*N*_*1*_*n*_*2*_*n*_*2*_*n*_*3*_*n*_*3*_ [40]. *N*_*1*_ locus is controlled by *MYB25*-like (aka, *MML1_A12*) gene on A12 chromosome, which generates small interfering RNAs from its 3′ end that post-transcriptionally silence both homoeologs of this gene [41], and *n*_*2*_ locus is related to *MYB25*-like gene on homoeologous D12 chromosome [37, 42]. These three cotton lines were chosen on the basis of their distinct fiber characteristics: GhTM-1 has both lint and fuzz fibers, Gb3–79 has almost exclusively lint fibers, and GhMD17 is fiberless (Fig. 1a). Both GhTM-1 and Gb3–79 have similar lint fiber properties in mature seeds, except for the lack of fuzz fiber on the Gb3–79 seed [42]. The fuzz fiber varies by genotypes and present in the Upland cotton (*G. hirsutum*) but largely absent in Pima cotton (*G. barbadense*). The genetic basis of this difference is not fully understood and is likely related to mutation of *MYB25*-like on homoeologous D12 chromosome [42].

During fiber initiation stage (0 and 2 DPA), both GhTM-1 and Gb3–79 had fibers of ~ 10 μm in length at 0 DPA and ~ 50 μm at 2 DPA, with little difference between GhTM-1 and Gb3–79, although fiber growth was slightly faster in Gb3–79 than in GhTM-1 (Fig. 1c). GhMD17 lacked any visible fiber cell initials during those stages (Fig. 1b). Thus, GhTM-1 and Gb3–79 have a similar fiber initiation process, which is blocked in GhMD17.

### RNA-seq analysis of fiber and epidermal cells separated by LCM

In wild-type ovules at 0 DPA and fiber-less mutants at both 0 and 2 DPA, LCM was used to cut the top layer of epidermal cells. For wild-type ovules at 2 DPA, LCM was used to cut along the epidermal surface to collect protruding fibers, followed by a second cut to harvest underlaying epidermal cells (Fig. 1d and Supplemental Figure 1a). LCM materials in each sample with two biological replicates were used for total RNA extraction (Supplemental Figure 1b), library construction, and sequencing. Sequencing reads were mapped onto the *G. hirsutism* genome v2.0 (GhTM-1 and GhMD17) and *G. barbadense* genome v1.0 (Gb3–79) [4] (Supplemental Table 1), and read counts were normalized to fragments per kilobase per million (FPKM) for gene expression analysis (see Methods).

**Fig. 2:**
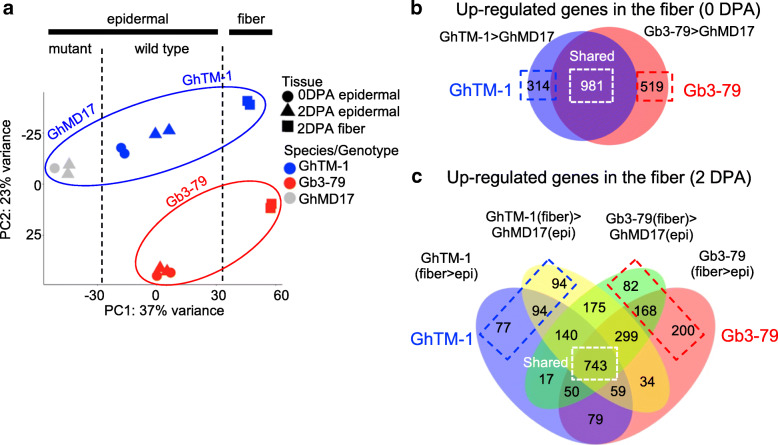
Gene expression divergence between tissue types and between species. **a** Principal component analysis of gene expression data from the LCM samples. Gene expression of GhTM-1 (blue), Gb3–79 (red), GhMD17 (gray) are separated by tissue types (dotted lines) and by species (oval circles) in a combination of developmental stage and tissue: epidermal at 0 DPA (circle), epidermal at 2 DPA (triangle), and fiber at 2 DPA (square). **b** Venn Diagram analysis of upregulated genes in fiber cell initials at 0 DPA; GhTM-1 > GhMD17 (light blue), and Gb3–79 > GhMD17 (pink), including those shared (white box) and specific to GhTM-1 (blue) or Gb3–79 (red) (dashed boxes). **c** Venn Diagram analysis of upregulated genes in early fiber cells at 2 DPA; GhTM-1 (fiber>epi) (light blue), Gh3–79 (fiber>epi) (pink), GhTM-1(fiber) > GhMD17(epi) (yellow), and Gb3–79(fiber) > GhMD17(epi) (green), including those shared (white box) and specific to GhTM-1 (blue) or Gb3–79 (red) (dashed boxes)

Principal component analysis (PCA) showed a clear separation of gene expression variance by tissue types (PC1: 37% of variance), followed by species (PC2: 23% of variance) (Fig. 2a). Epidermal samples between the 0 and 2 DPA stages were not separated. We also determined the Pearson’s correlation coefficient of all 8 samples (averaged values of two biological replicates) and performed hierarchical clustering analysis among these samples (Supplemental Figure 2a). This revealed three major groups: fiber samples (GhTM-1 and Gb3–79 at 2 DPA), mutant epidermal samples (GhMD17 at 0 and 2 DPA), and Gb3–79 epidermal samples (0 and 2 DPA). GhTM-1 epidermal sample at 0 DPA was grouped with Gb3–79 epidermal samples, while GhTM-1 epidermal sample at 2 DPA and mutant epidermal samples were in the same group. These results indicate that gene expression differences between tissue types and between species of LCM samples were suitable for this study.

At 0 DPA, fiber cell initials develop in the wild type but not in the mutant. To identify differentially expressed genes in fibers at 0 DPA, we compared expression differences in the epidermal samples between the wild type (GhTM-1 and Gb3–79) and mutant (GhMD17). We identified 1295 and 1500 upregulated genes in GhTM-1 and Gb3–79 fiber cell initials, respectively, 981 of which (> 60%) were shared in both, while 314 and 519 were specific to GhTM-1 and Gb3–79, respectively (Fig. 2b, Supplemental Tables 2 and 3). In addition, 2101 and 2582 genes were down-regulated in GhTM-1 and Gb3–79 fiber cell initials, respectively, 1528 of which (> 60%) were shared in both (Supplemental Figure 2b, Supplemental Table 2).

In epidermal cells, there were 291 and 181 upregulated genes in GhTM-1 and Gb3–79, respectively, at 0 DPA (Supplemental Figure 3a and Supplemental Table 3a), and 583 and 409 upregulated genes in GhTM-1 and Gb3–79, respectively, at 2 DPA (Supplemental Figure 3b and Supplemental Table 3a). The number of shared genes between GhTM-1 and Gb3–79 was relatively small, only 14 genes (~ 5%) at 2 DPA and 121 genes (~ 20%) genes at 2 DPA, suggesting expression divergence in epidermal layer cells between Upland and Pima cotton. GO analysis showed overrepresentation of upregulated genes at 2 DPA in lipid metabolic process (*p*-value 0.00045) and DNA replication (*p*-value 2.7e-12), consistent with biological processes involved ovule and seed development.

At 2 DPA, we compared gene expression of LCM fiber cell with epidermal cell tissues within the same genotype (GhTM-1 or Gb3–79) and between the fiber cell (GhTM-1 and Gb3–79) and epidermal cell tissues in the fiber-less mutant (GhMD17). We identified 1861 and 2046 up-regulated genes in GhTM-1 and Gb3–79 fiber cells, respectively, 743 of which (> 35%) were shared in both, while 265 and 450 were unique to GhTM-1 and Gb3–79, respectively (Fig. 2c, Supplemental Tables 3 and 4). We also identified 2978 and 4269 down-regulated genes in GhTM-1 and Gb3–79 fiber cells, respectively, and 1626 of which (> 35%) were shared in both (Supplemental Figure 2c, Supplemental Table 4).

**Fig. 3:**
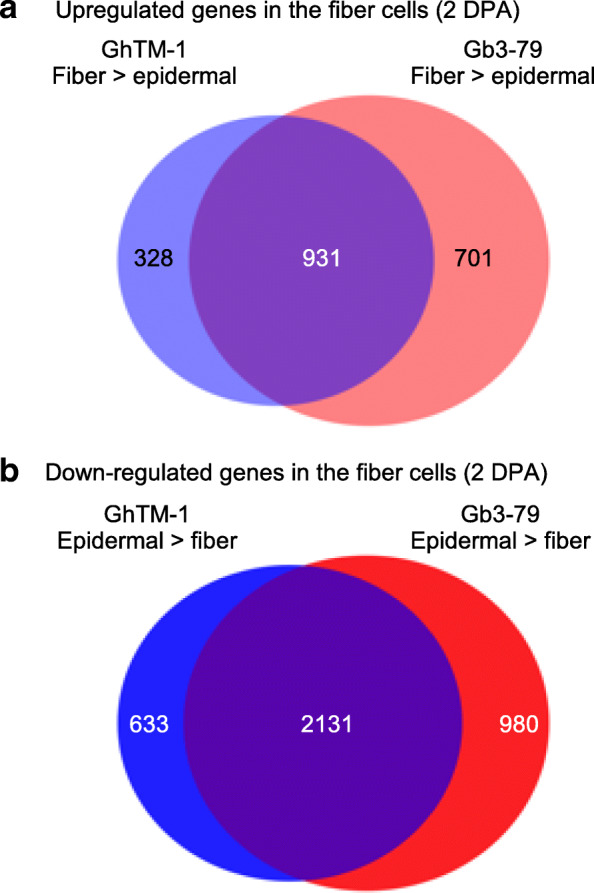
Gene expression divergence between fiber and epidermal cells at 2 DPA. **a** Venn Diagram analysis of upregulated genes in fiber cells at 2 DPA; GhTM-1 fiber > epidermal (light blue) and Gb3–79 fiber > epidermal (pink). **c** Venn Diagram analysis of down-regulated genes in fiber cells at 2 DPA; GhTM-1 epidermal > fiber (blue) and Gb3–79 epidermal > fiber (red)

To exclude a possibility of the fiberless mutant (MD17) effect, we generated data between GhTM-1 and Gb3–79 in fiber and epidermal samples at 2DPA (Fig. 3 and Supplemental Table 4a). A total of 1259 and 1631 genes in fiber cells at 2 DPA were upregulated in GhTM-1 and Gb3–79, respectively (Fig. 3a), while 2764 and 3111 genes in epidermal cells were upregulated in GhTM-1 and Gb3–79, respectively (Fig. 3b). Among them, more upregulated genes were shared between GhTM-1 and Gb3–79 in epidermal layer (2131, > 68.5%) than in fiber (931, > 56.8%), which were consistent with the results including GhMD17 epidermal cells. These differentially expressed genes in the fiber cell tissues were used for further analysis.

### Dramatic gene expression changes in early stages of cotton fiber cell development

**Fig. 4:**
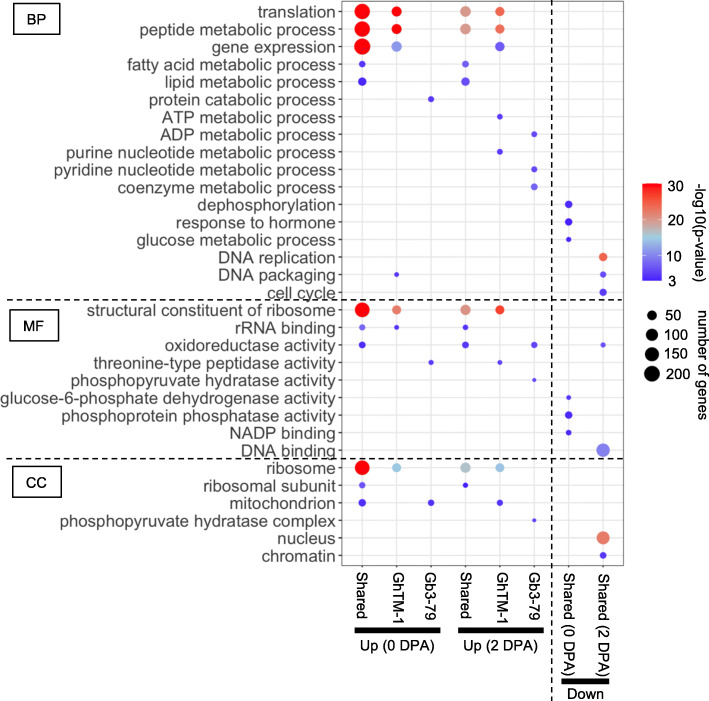
Gene ontology (GO) analysis of upregulated and down-regulated genes in early fiber cells. GO term overrepresentation of upregulated (Up), down-regulated (Down), or shared genes in GhTM-1 and Gb3–79 in early fiber cells at 0 or 2 DPA in Biological Process (BP), Molecular Function (MF), and Cell Components (CC). Color bar and circle size represent log10(significant *p*-value) and number of genes, respectively

Gene Ontology (GO) analysis of these differentially expressed genes showed a consistent overrepresentation of translation (ribosome activity), gene expression, and peptide, lipid, and fatty acid metabolic processes in the upregulated genes in fibers (Fig. 4 and Supplemental Table 4b). These results may indicate that ribosome biosynthesis, required for protein synthesis as well as lipid and fatty acid metabolism, were active during fiber cell initiation. Moreover, the upregulated genes between the species were overrepresented in ATP and purine metabolic processes in GhTM-1, and ADP and pyridine (similar structure of pyrimidine) metabolic processes were overrepresented in Gb3–79. ATP is a critical energy resource for fiber growth. In cultured cotton ovules, fibers release ATP as they grow, and when the ectoapyrase activity is blocked by the addition of polyclonal anti-apyrase antibodies or small molecule inhibitors, the medium ATP level rises and fiber growth is suppressed; low concentrations of hydrolyzable nucleotides ATPgammaS/ADPbetaS stimulate fiber growth [43]. Thus, the difference of ATP and ADP metabolic processes between GhTM-1 and Gb3–79 may contribute to longer fibers in Gb3–79.

Among down-regulated genes in fibers, they were overrepresented in response to hormones and glucose metabolic process at 0 DPA and DNA replication and the cell cycle at 2 DPA. Together, these data may suggest that cell cycle and DNA replication have attenuated in fiber cells, which require active ribosome biosynthesis for translational regulation in fiber cells. Down-regulation of phytohormone-related genes in the fiber cells may suggest upregulation of these genes in ovules to support fiber elongation.

### Pervasive homoeolog expression bias in fiber and epidermal cells

Allotetraploid cottons consist of A and D subgenomes (A and D) with 52,514 orthogroups representing 15,220 pairs of A and D homeologs in *G. hirsutum* and *G. barbadense* (Supplemental Tables 5 and 5a). Among them, ~ 20% of homeolog pairs were differentially expressed (Supplemental Figure 4a), with 10% more D homoeologs (1441-1550) expressed at higher levels (D > A) than the A homoeologs (1250-1402, A > D) in both fiber and epidermal cells (Supplemental Figure 4b,c). These data are consistent with the notion of dramatic gene expression divergence between the homoeologs, despite gene numbers and synteny are conserved between the A and D subgenomes in the allotetraploid cottons [4]. This trend of biased expression is shared by 251 and 81 homoeolog pairs that encode cell cycle regulation genes and ribosomal protein subunit genes, respectively, with expression bias for ~ 20% of homeolog pairs involved in cell cycle regulation and for ~ 15% of homoeologs encoding ribosomal protein subunits (Supplemental Figure 4d). Interestingly, for cell cycle regulation genes, 10% more D than A homoeologs had biased expression, whereas this number of homoeologs with expression bias was increased to 40–50% more in D than in A homoeologs of ribosomal protein subunit genes (Supplemental Figure 4d). This high number of homoeologs with expression bias in the early stages of fiber cells may indicate that deep subfunctionalization of homoeologous genes during active developmental process of fiber cell initiation and elongation.

### Translational regulation in early cotton fiber cell development

During fiber cell development, cell cycle is predicted to attenuate in 20–30% of epidermal cells, and those cells become fiber cell initials. Cell cycle arrest in a cell often leads to active ribosome biosynthesis [44]. This is consistent with developmental process of fiber cell initiation, when the arrest of the cell cycle in the epidermal cells induces active ribosome biosynthesis for fiber cell initiation and elongation.

**Fig. 5:**
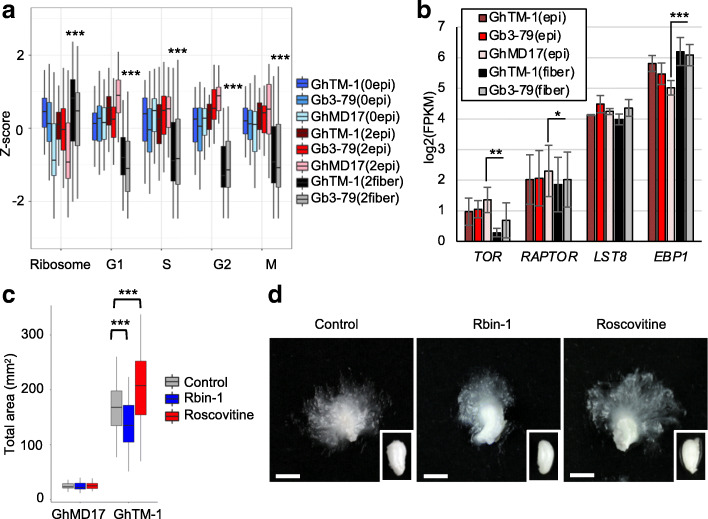
Effects of ribosome biosynthesis and cell cycle inhibitors on fiber cell development. **a** Box plots indicating overall gene expression change (z-score) in ribosome protein subunit genes and cell cycle related genes (G1-M). Box colors indicate samples of epidermal at 0 DPA (blue), epidermal at 2 DPA (red), and fiber at 2 DPA (black) of GhTM-1 (dark colored), Gh3–79 (intermediate colored), and GhMD17 (light colored). Three asterisks indicate a statistical significance level of *P* < 0.001 (Student *t*-test). **b** Bar plots indicate gene expression levels (FPKM) of *TOR*, *RAPTOR*, *LST8*, and *EBP1* in 2 DPA samples. Colors are identical to those in (**a**). One, two, and three asterisks indicate statistical significance levels of *P* < 0.05, < 0.01, and < 0.001, respectively (Student *t*-test). **c** Total area (mm^2^) of ovules of GhTM-1 and GhMD17 incubated in culture for 21 days with control (grey), Rbin-1 (blue), and Roscovitine (red) treatments. Three asterisks indicate a statistical significance level of *P* < 0.001 (Student’s *t* test). **d** Photos of typical ovules with fibers in GhTM-1 and GhMD17 ovules (insets, right bottom) incubated with mock (control, left), Rbin-1 (middle), and Roscovitine (right). Bars = 10 mm

Arabidopsis has 231 annotated genes for cell cycle regulation, and cotton has a total of 845 putative cell-cycle regulation genes, including 366 involved in G1, 283 in G2, 122 in S, and 74 in M stages (Supplemental Table 6). Approximately 85% of all putative cell-cycle regulation genes were down-regulated in fiber cells (Fig. 5a and Supplemental Figure 5a). Cotton has 681 annotated genes encoding for ribosome protein subunits, consisting of large subunit, RPL and small subunit RPS, respectively (Supplemental Table 6). These multiple putative ribosomal subunit genes (618 genes) are grouped into 121 putative ribosomal proteins. In contrast to down-regulation of cell cycle related genes, approximately 75% of putative ribosomal protein genes were up-regulated, while 8% were down-regulated (Supplemental Figure 5b). Plants have more ribosomal protein genes than animals or yeasts [45], and many of those paralogues are differentially expressed during development and in UV-B responses [46]. For example, in *Arabidopsis RPL16A* and *RPL16B* are expressed in developing pollen and early phase of lateral root initiation [47], a process similar to fiber cell initiation. *AtRPS5A* gene is strongly expressed in dividing cells, whereas *AtRPS5B* expression correlates with cell differentiation rather than cell division [48]. These results suggest that during fiber cell initiation, cell cycle was attenuated, and ribosomal protein biosynthesis is activated and may regulate tissue-specific expression in fiber and epidermal cells.

Cell cycle is regulated by Target of Rapamycin Complex1 (TORC1), which consists of three proteins *TARGET OF RAPAMYCIN* (*TOR*), *RAPTOR*, and *LETHAL WITH SEC THIRTEEN PROTEIN 8* (*LST8*) [49, 50]. *ErbB-3 EPIDERMAL GROWTH FACTOR BINDING PROTEIN1* (*EBP1*) is a TOR target. TORC1 is also a key regulator of many other biological processes such as DNA and RNA synthesis and autophagy and is conserved among plants and animals [51]. In cotton, *TOR*, *RAPATOR*, *LST8*, and *EBP1* have multiple members, two homoeolog pairs each for *TOR*, *RAPTOR*, and *EBP1*, and one homoeolog pair for *LST8*. Expression levels among multiple members of each gene were similar and averaged for the analysis (Supplemental Table 7). In fiber cells at 2 DPA, *TOR* was down-regulated in both GhTM-1 and Gb3–79 (Fig. 5b), while *RAPTOR* and *LST8* expression levels were similar to epidermal cells, and *EBP1* was slightly up-regulated. In *Arabidopsis*, *TOR* is expressed in the primary meristem, embryo, and endosperm, but not in differentiated cells [52], while the mutant is embryonically lethal. Notably, Arabidopsis *EBP1,* a homolog of human *EBP1* for ribosomal protein biosynthesis, is required for expression of cell cycle genes and regulates cell proliferation and size in plants [53]. Thus, in fiber cells down-regulation of *TOR* may inhibit the cell cycle, and strong expression of *EBP1* could promote ribosome biosynthesis for fiber cell expansion. Interestingly, both cell cycle regulators *TOR* and *RAPTOR* have been identified as epialleles, which are demethylated in the cultivated cotton species but not in the wild relatives [39].

To further test roles of ribosome biosynthesis and cell cycle regulation in fiber cell development, we used pharmacological assays in cultured GhTM-1 and GhMD17 ovules. Ribozinoindoles (Rbin-1) are potent and reversible triazinoindole-based inhibitors of eukaryotic ribosome biogenesis [54], and Roscovitine is a cyclin-dependent kinase inhibitor for cell cycles [55]. Cotton ovules were isolated from fertilized flowers of GhTM-1 and GhMD17 at 0 DPA and incubated in BT medium for 21 days following a published protocol [43], with or without inhibitors. Inhibiting cell cycle by Roscovitine (2 μg/L) increased total fiber area and length at statistically significant levels, whereas impeding ribosome biosynthesis by Rbin-1 (40 μg/L) reduced the fiber area (Fig. 5c, d), and the ovule size was not affected by either treatment (Fig. 5d). Indeed, chemical inhibitors had no effects on ovule development in the GhMD17 that is fiberless (Fig. 5c left). The altered fiber area could result from increased fiber length, number, or both. To discern this, we further measured the number or density of fiber cell initials after 2 days of each treatment (Supplemental Figure 6). The result showed a similar trend of increasing and reducing fiber cell density by Roscovitine and Rbin-1 treatments, respectively. These results support the hypothesis that cell cycle attenuation and active ribosome biosynthesis promote fiber cell initiation and elongation.

### Dynamic roles of phytohormones and MYB transcription factors in early fiber cells

**Fig. 6:**
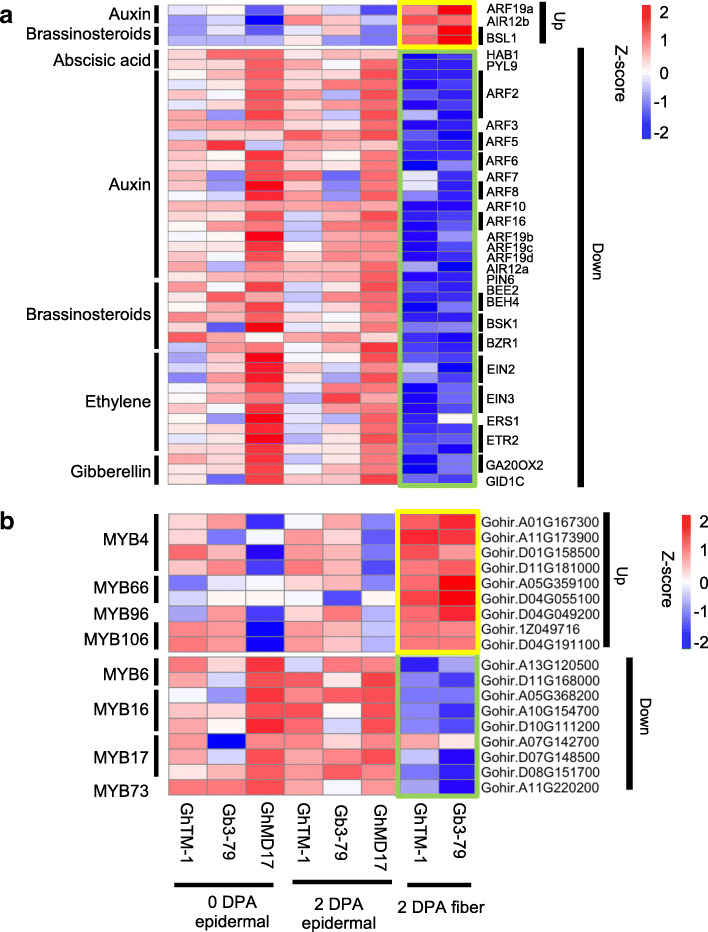
Differential expression of the genes encoding phytohormone pathways and MYB transcription factors. **a** Heatmap showing phytohormone related genes that are upregulated (top) and down-regulated (bottom) during ovule and fiber development with epidermal at 0 DPA (left), epidermal at 2 DPA (middle), and fiber at 2 DPA (right) in GhTM-1, Gh3–79, and GhMD17. Names of *G. hirsutum* phytohormone related genes (right) are predicted based on Arabidopsis homologs. Box colors represent up-regulation (yellow) and down-regulation (green) of genes in early fiber cells. **b** Heatmap showing MYB transcription factor related genes that are upregulated (top) and down-regulated (bottom) during ovule and fiber development with epidermal at 0 DPA (left), epidermal 2 at DPA (middle), and fiber 2 at DPA (right) in GhTM-1, Gh3–79, and GhMD17. Box colors represent upregulation (yellow) and down-regulation (green) of genes in early fiber cells

Phytohormones play important roles in fiber cell development [2, 25], but the temporal regulation of gene expression in early fiber developmental stages is poorly understood. Using LCM, we unexpectedly uncovered genome-wide down-regulation of 43 phytohormone related genes with only a few up-regulated genes in early fiber cells (Fig. 6a and Supplemental Table 8). This is probably because most genes are upregulated in the epidermal layer cells that support fiber cells. Abscisic acid (ABA) is known to inhibit fiber cell initiation and elongation [56]. ABA in ovules at 0 DPA accumulates at much higher levels in the *Ligon-lintless 1* mutant than in the wild type [57]. This is consistent with the down-regulation of key regulators in the ABA pathway (Fig. 6a), including phosphate type-2C *HAB1* (a homeolog of *ABA INSENSITIVE 1*, *ABI1*; and *ABI2*) [58] and ABA receptor gene *PYRABACTIN RESISTANCE-LIKE PROTEIN 9* (*PYL9*) (known to interact with *ABI1* and *ABI2*) [59]. Down-regulation of the ABA regulator and receptor genes may promote fiber cell initiation and elongation.

Gibberellins promote fiber cell elongation and increase ABA and auxin levels during fiber development to enhance fiber quality (strength) [60]. Over-expression of gibberellin biosynthesis gene *GIBBERELLIN 20-OXIDASE1* (*GA20OX1*) leads to more and longer fibers [61], while their homeologs *GA20OX2* and *GA20OX3* are largely expressed in the ovule. Consistently, *GA20OX2* was upregulated in the ovule and down-regulated in fibers (Fig. 6a).

Ethylene regulates fruit ripening and inhibits vegetative growth [62, 63], and is reported to promote fiber elongation in cotton, which is mediated by very-long-chain fatty acid signaling pathway [29]. However, the mechanism for ethylene in fiber initiation remains unclear. We found down-regulating of the ethylene signaling pathway genes like *ETHYLENE INSENSITIVE 2* (*EIN2*) and *EIN3* in fibers, which may suggest a negative role for ethylene in fiber cell initiation (Fig. 6a).

Auxin promotes fiber initiation and accumulates in ovules prior to fertilization [10]. Auxin is transported from ovule to fiber cells during fiber cell development [26]. Consistent with this notion, most genes encoding AUXIN RESPONSE FACTORs (ARFs) and PIN-FORMED6 (PIN6), a key auxin transporter, were upregulated in the ovules and down-regulated in early fiber cells (Fig. 6a). This is reminiscent of the finding that *ARF2*, *ARF18* and *PIN6* were expressed in ovules in the fiber initiation stage [60]. Interestingly, although the majority of among *ARF* gene family members are upregulated in the ovules, a few such as *ARF19a* and *ARF19b* homoeolog pair, showed opposite expression patterns in the fiber cells (Supplemental Figure 7), suggesting functional divergence between homoeologs for fiber cell development in polyploid cotton.

Brassinosteroids promote both fiber cell initiation and elongation [28, 64]. A key regulator of brassinosteroid signaling, *BRASSINAZOLE-RESISTANT 1* (*BZR1*), induces cotton fiber development [65]. *BSU1* is a dominant suppressor of *BRI1* and is expressed in elongating cells in Arabidopsis [66]. In cotton fiber cells, *BSU1* was upregulated, whereas *BZR1* and its semidominant suppressor gene (*BES1*) [67] were down-regulated (Fig. 6a), suggesting a role for brassinosteroids in fiber cell initiation.

In addition to phytohormones, MYB transcription factors regulate fiber cell development in seeds and trichome development in leaves [37, 38, 68]. Among 18 MYB transcription factor genes examined, nine were upregulated, and the other nine were down-regulated in early fiber cells (Fig. 6b and Supplemental Table 8). The upregulated genes included *GhMYB25*, *GhMYB109*, and cotton homologs of Arabidopsis *MYB4*, *MYB66*, *MYB96*, and *MYB106* (Supplemental Table 8). Notably, cotton homologs of *MYB106* (*GhMYB25* in cotton) were up-regulated, while cotton *MYB16* homologs were down-regulated in fibers (Fig. 6b), while Arabidopsis *MYB16* and *MYB106* are in the same clade (Supplemental Figure 8). Similarly, cotton *MYB4* was upregulated, and *MYB6* was down-regulated in early fiber cells (Fig. 6b and Supplemental Figure 8), but they are closed related MYB genes. These results may suggest functional divergence between closely related homologous genes during fiber cell development.

## Discussion

**Fig. 7:**
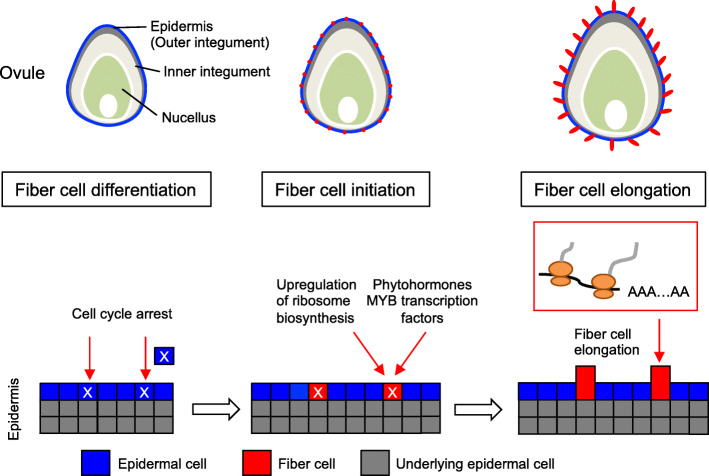
A model of cotton fiber cell initiation and elongation. Fiber cell differentiation (left): Cell cycle arrest occurs in a subset of epidermal cells that are differentiating into fiber cells (blue boxes marked by X). Fiber cell initiation (middle): Upregulation of putative ribosomal protein subunit genes induces ribosome biosynthesis in early fiber cells (red boxes marked by X). Fiber cell elongation (right): Increased ribosome activities may promote translation for fiber cell elongation (red elongated boxes). Developing ovules corresponding to developmental stages (from left, middle, to right) are shown above corresponding stages

In this study, we used LCM to successfully dissect fiber and epidermal cells from the cotton ovules during fiber cell initiation and investigated gene expression changes between fiber cell initials and epidermal layer cells. Our data support a new model of fiber cell initiation and elongation, involving regulation of cell cycle and ribosome biosynthesis activities, as well as phytohormones and MYB transcription factors (Fig. 7).

Fiber cells are initiated in 20–30% of epidermal cells at or prior to 0 DPA and before fertilization. Fiber cell initials differentiate from epidermal layer cells and involve a rapid and dramatic transition from actively dividing ovular cells to elongation of new singular fiber cells. At the cellular and molecular levels, cell cycle is predicted to arrest and coupled with active biosynthesis of ribosome for translational activities including protein synthesis. This rapid process of cell differentiation is similar to the origin of human tumor cells, where cell cycle arrest and ribosome biosynthesis are intrinsically correlated [23, 24]. The tumor suppressor p53 has been shown to induce cell-cycle arrest in response to active ribosome biogenesis for rapid cell growth [23], and the transition associated with development and tumor metastasis is accelerated by upregulation of ribosome biogenesis during G1/S arrest [24]. By this analogy, although the cause and consequence are unknown, some unknown factor(s) may trigger cell cycle arrest in pre-fiber cells to induce active ribosome biosynthesis to prepare for rapid cell initiation and elongation. Considering that each fiber cell does not divide, abundant ribosomes may promote translational regulation and protein synthesis, leading to rapid fiber cell elongation.

The TOR signaling pathway integrates nutrient, hormone, growth factor and environmental inputs to control cell cycle, proliferation, metabolism, and growth in diverse organisms, including plants [51]. Loss of *TOR* function results in cell cycle arrest in G1 phase in human and yeast [69], which is consistent with the observation that cotton fibers cells are arrested at G1 stage [14, 70]. We found in early stages of fiber cells, *TOR* expression is downregulated (Fig. 5), which may induce ribosome biosynthesis and translation rate through the TOR signaling pathway [49]. Moreover, *EBP1* is upregulated in the cotton fiber cells, which can positively regulate ribosome biosynthesis as part of the TOR signaling pathway and auxin regulation in plants [53]. It is likely that the TOR signaling pathway may regulate cell cycle and ribosome biosynthesis in the ovule epidermis for fiber cell initiation as well as elongation in early fiber cells.

Phytohormones are essential for fiber cell initiation and development [2, 25, 56]. While some phytohormones such as ABA and cytokinin have a negative role in fiber cell development, many others such as auxin, gibberellins, and brassinosteroids positively regulate fiber cell initiation and development. It is unexpected to find genome-wide down-regulation of phytohormone related genes such as *ARFs*, *PIN6*, *BRZ1*, *EIN2*, *EIN3*, and *GA20OX2* in early fiber cells. This result may suggest an important role for spatial and temporal action of phytohormones in fiber cell development, which is revealed by the LCM approach in this study. Many phytohormone related genes such as *ARFs* and *PIN6* are strongly expressed in epidermal layer cells [60] (Fig. 6). Auxin may actively accumulate in the ovules and is transported into young fiber cells, reflecting a spatial and temporal role of auxin in fiber cell initiation. Indeed, expression of cotton *ARF2:GU*S in Arabidopsis is localized in the trichome base but not in the body or tip [71], consistent with its spatial and temporal role in leaf trichome development as in cotton fiber cell initiation. Alternatively, there may be crosstalk between phytohormone pathways and other factors such as cell cycle regulation and ribosome biosynthesis to regulate fiber cell development. For examples, inhibiting TOR signaling pathway decreases ABA synthesis [72], and auxin activates TOR signaling pathway and promotes translational regulation [73] and some other associated pathways [74].

MYB transcription factors are required for the development of leaf trichomes as well as seed hair or fiber cells [33–36]. Cotton has two major groups of MYB transcription factor genes, one expressed in trichomes or leaves and another largely in ovules and fiber cells [38]. Up and down-regulation of MYB transcription factor related genes may reflect different roles of these genes in trichome and fiber cell development in cotton. In Arabidopsis, *MYB16* and *MYB106* (*GhMYB25* in cotton) are closely related genes that promote cuticle formation in the epidermal cells including trichomes [75] and promote both trichome branching and expansion [76], while the cotton homolog GhMyb25 regulates cotton fiber cell development [19]. Thus, upregulation of *MYB106* and down-regulation of *MYB16* in early fiber cells may reflect their functional divergence in trichome and fiber cell development. This expression divergence between closely related homoeologs is common to many other genes including those in phytohormone, cell cycle, and ribosome biosynthesis pathways. The mechanism for this expression divergence may involve small RNAs including siRNAs and miRNAs, DNA methylation, and other epigenetic modifications [20–22, 34].

Finally, the genes involved in photoperiodic flowering and cell cycle regulation such as *TOR* and *RAPTOR* are reported to be epialleles, which are demethylated and highly expressed in the cultivated cotton species but not in the wild relatives [39]. This result underscores important role for epigenetic regulation in selection and domestication of agronomic traits including fiber cell development, possibly fiber yield and quality. Our study has provided valuable genomic resources and new insight into roles of cell cycle regulation, ribosome biosynthesis, and spatial-temporal action of phytohormones and MYB transcription factors in early stages of fiber cell development. Understanding this complex regulation during fiber cell initiation and elongation may help us develop new breeding and biotechnological tools to improve cotton fiber yield and quality.

## Methods

### Plant materials

Two cultivated species GhTM-1 (*G. hirsutum*) and Gb3–79 (*G. barbadense*), and the fiber-less mutant GhMD17 (*G. hirsutum* background) were grown in soil in a greenhouse under natural sun light at The University of Texas at Austin. Flowers of cotton were tagged on the day of (post) anthesis (0 DPA) and collected at 1–3 pm to minimize diurnal effects. Immediately after flower collection, ovules were manually dissected from flowers under a dissection microscope. Flowers were collected in March 2016 for LCM RNA-seq analysis and in August 2018 for ovule culture assays. Unless noted otherwise, three biological replicates were used for each analysis.

### Image analysis of cotton ovules and fiber cells

Cotton ovules were fixed with FAA solution (3.7% formaldehyde, 5% acetic acid, and 50% ethanol). They were then processed using an ethanol dilution series (50,70, 90, 95, and 100%), followed by an xylene:ethanol series (1:3, 1:1, 3:1, and 1:0), and finally multiple changes in a paraffin solution to embed siliques. Paraffin-embedded tissues were sectioned at 7 μm by microtome and mounted on glass side. Slides were deparaffinized using a brief xylene bath. Photos of ovules were taken using a light microscope ECLIPSE Ni (Nikon, Tokyo, Japan) and processed with ImageJ (https://imagej.nih.gov/ij/).

### Laser-capture microdissection (LCM) and mRNA-seq library preparation

Ovules were collected from flowers at 0 and 2 DPA from GhTM-1, Gb3–79, and GhMD17, each with two biological replicates due to the laborious process, as performed in other studies using microarrays [77] and RNA-seq [78]. Tissue fixation was performed as described in [79]. Briefly, cotton ovaries were cut using a razor, and individual ovules were removed using forceps, fixed in a solution containing 75% ethanol and 25% acetic acid under vacuum for 15 min, and incubated at 4 °C for overnight. The samples transferred into fresh 75% ethanol and 25% acetic acid for 15 min on ice and microwaved at 250 W for 15 min using a PELCO BioWave (TED PELLA Inc., Redding, CA); the process was repeated three times each with a new solution. The samples were then dehydrated by ethanol series (70, 80, 90, 100, and 100%) with microwaving at 58 °C, 300 W, for 1.5 min at each step. Samples were then transferred to butanol in two steps (50%:50% ethanol:butanol followed by 100% butanol, each time microwaved at 58 °C, 300 W, for 1.5 min. Finally, samples were transferred into molten paraffin (50%:50% butanol:melted paraffin, then 100% molten paraffin, with microwaving at 58 °C, 250 W, for 10 min for each step). Molten paraffin was changed four times, with the sample vials immersed in a hot water bath, and microwaved at 58 °C, 250 W, for 30 min after each change. Finally, the samples were embedded in paraffin tissue blocks. Paraffin-embedded tissues were sectioned by a microtome (at 7 μm) and mounted on a pen-foil slide (ZEISS, Oberkochen, Germany). Fiber and epidermal cells were isolated and collected using Zeiss PALM Laser Microdissection with AdhesiveCap 500 (ZEISS, Oberkochen, Germany). The LCM fiber and epidermal tissues were subject to RNA extraction by RNA Aqueous Micro Total RNA Isolation Kit (Thermo Fisher Scientific, Waltham, MA), following the manufacturer’s protocol. The total RNA was treated with DNase treatment and qualified by a Bioanalyzer (Agilent, Santa Clara, CA) with RNA Integrity Number (RIN) > 6. An aliquot of total RNA (~ 100 ng) was used for RNA-seq library construction using the NEB Next Ultra RNA Library Prep Kit (New England Biolabs, Ipswich, MA). mRNA-seq libraries were sequenced by paired-end reads (150 bp) using an Illumina HiSeq 2500 (Illumina, San Diego, CA).

### mRNA-seq data analysis of LCM samples

LCM mRNA-seq reads were mapped to *G. hirsutum* TM-1 genome v2.0 (GhTM-1 and GhMD17) and *G. barbadense* genome V1.0 (Gb3–79) whole genome [4] using Tophat 2.1.1 [80]. Uniquely mapped reads were extracted and analyzed by Cufflinks 2.2.1 [81] to determine gene expression levels using Fragments Per Kilobase Million (FPKM). Ortholog tables can be downloaded from Phytozome (https://genome.jgi.doe.gov/portal/pages/dynamicOrganismDownload.jsf?organism=Ghirsutum with the file name: /Ghirsutum/v2.1/orthology/Ghirsutum/inparanoid_Ghirsutum_527_v2.1.tar.gz/inParanoid_Ghirsutum_527_v2.1_Gbarbadense_526_v1.1.) (Supplemental Table 5a). Orthologs of *G. hirsutum* and *G. barbadense* (52,514 genes) expressed in these samples were used for further analysis. Values from two biological replicates were averaged to determine differentially expressed genes (DEGs) using the criteria of > 5 FPKM, ANOVA (*P* < 0.01), and fold changes > 1.5 (wild type epidermal vs. mutant epidermal; fiber vs. wild type epidermal) or > 2 (fiber vs. mutant epidermal).

Principal component analysis (PCA) was performed using log2-transformed gene expression values with singular value decomposition via the prcomp function in R (https://www.r-project.org).

Averaged gene expression values from two biological replicates were used for correlation coefficiency analysis. Pearsons’s correlation coefficients were determined using the *cor* function in R (https://www.r-project.org).

Gene Ontology (GO) analysis for upregulated and down-regulated genes was performed using the Bioconductor package topGO (https://bioconductor.org/packages/release/bioc/html/topGO.html) to identify overrepresented GO terms with statistical significance (*P* < 0.0001).

Expression values of all 15,220 homoeolog pairs were divided into seven groups as previously described [4]: no expression (FPKM = 0 in both A and D), A = D (FPKM > 1 in both A and D, A < 2xD and D < 2xA), low expression (FPKM< 1 in both A and D), D = 0 (A FPKM> 1 and D = 0), A = 0 (D FPKM> 1 and A = 0), D > A (D is FPKM> 1 and D > 2xA), and A > D (A is FPKM> 1 and A > 2xD).

For phylogenetic analysis, protein sequences were obtained from Phyotozome using BIOMART, aligned using Clustal Omega [82] at EMBL-EBI (https://www.ebi.ac.uk/Tools/msa/clustalo/); the alignments were trimmed, and a neighbor-joining trees generated using JalView (http://www.jalview.org). Trees were visualized using Bioconductor package ggtree [83].

### Ovule culture

Ovules were collected from fertilized flowers at 0 DPA of GhTM-1 and GhMD17. Ovule culture was conducted using a modified protocol as previously described [43]. Briefly, ovaries were isolated from flowers and surface sterilized in 100% ethanol for 15 min, and further dissected to expose the ovules. Individual ovules were collected and floated in BT medium [56] with or without 40 μg/L Rbin-1 (Axon Medchem, Groningen, Netherlands) [54] or 2 μg/L Roscovitine (#R7772-1MG, MilliporeSigma, Burlington, MA) [55] under dark conditions at 28 °C. For fiber initialization density counts, after 2 days, cultured ovules were briefly dried on a laboratory wipe before being gently placed on a microscope slide covered a UV curable adhesive (All-Ways Adhesives, Bensenville, IL). These slides were exposed to a hand-held UV torch for 30 s to set the adhesive before removing the ovules, and the resulting impressions were imaged via stereomicroscope SMZ1500 (Nikon, Tokyo, Japan). Fiber density was calculated by defining a region of interest in ImageJ, measuring the area and counting the fiber cell impressions.

For area measurements, cultured ovules were incubated for 21 days and then transferred to a glass plate where a stream of distilled water from a lab wash bottle was used to gently splay fibers. Excess water was then removed using laboratory wipes, and photos were taken. Total fiber and ovule areas were calculated and analyzed using ImageJ (https://imagej.nih.gov/ij/).

## Supplementary Information

**Additional file 1: Supplemental Figure 1.** Images of Laser Capture Microdissection (LCM) and RNA quality in LCM samples. **a** Isolation of fiber and epidermal cell tissues by Laser Capture Microdissection (LCM) method. Top represents epidermal cell isolation from ovules at 0 DPA in GhTM-1 and Gb3–79, and GhMD17. Bottom shows separation of fiber and epidermal cell layers from ovules at 2 DPA in GhTM-1 and Gb3–79. Bar represents 150 μm. **b** Line plots showing RNA size (x-axis) and quantity (y-axis) measured by Bioanalyzer with RIN number in GhTM-1 epidermal cells (0 DPA) (left) and GhTM-1 fiber cells (2 DPA) (right). Two ribosomal RNA peaks are clearly visible. **Supplemental Figure 2.** Gene expression correlation between GhTM-1, Gb3–79, and GhMD17 and down-regulated genes in early fiber cells. **a** Pearson’s correlation coefficients of LCM RNA-seq samples averaged between 2 replicates. Hierarchical clustering was based on geometric distance. **b** Venn diagram analysis of down-regulated genes in early fiber cells at 0 DPA; GhMD17 > GhTM-1 (blue) and GhMD17 > Gb3–79 (red). Shared down-regulated genes in fibers at 0 DPA (white) between comparisons are shown in dashed box. **c** Venn diagram analysis of down-regulated genes in early fiber cells at 2 DPA; GhTM-1(epi > fiber) (blue), Gh3–79 (epi > fiber) (red), GhMD17(epi) > GhTM-1(fiber) (yellow), and GhMD17(epi) > Gb3–79(fiber) (green). Shared down-regulated genes in early fiber cells at 2 DPA (white) between comparisons are shown in dashed box. **Supplemental Figure 3.** Gene expression divergence between GhTM-1 and Gb3–79 in epidermal cells at 0 and 2 DPA. **a** Venn Diagram analysis of upregulated genes in epidermal cells at 0 DPA; GhTM-1 0epi > 2epi (light blue) and Gb3–79 0epi > 2epi (pink). **b** Venn Diagram analysis of upregulated genes in epidermal cells at 2 DPA; GhTM-1 2epi > 0epi (blue) and Gb3–79 2epi > 0epi (red). **Supplemental Figure 4.** Biased expression of A and D homoeologs in GhTM-1 and Gb3–79. **a** Number of A and D homoeologs (pairs), showing no expression (orange), A = D (red), low expression (FPKM< 1) (yellow), D = 0 (A FPKM> 1) (light green), A = 0 (D FPKM> 1) (green), D > A (D FPKM> 1 and D > 2xA) (blue), and A > D (A FPKM> 1 and A > 2xD) (light blue). **b** Number of A and D homoeologs (pairs), showing D = 0 (A FPKM> 1) (light green), A = 0 (D FPKM> 1) (green), D > A (D FPKM> 1 and D > 2xA) (blue), and A > D (A FPKM> 1 and A > 2xD) (light blue). **c** Number of A and D homoeologs with biased expression, either A-homoeologs highly expressed (red) or D-homoeologs highly expressed (blue). **d** Percentage of A and D homoeologs (pairs) with biased expression involved in cell cycle regulation (251 homeolog pairs) and ribosomal protein biosynthesis (81 homeolog pairs) between epidermal (0 and 2 DPA) and fiber cells. Colors represent A homoeologs (cell cycle, red; ribosome, pink) and D homoeologs (cell cycle, blue; ribosome, light blue). **Supplemental Figure 5.** Clustering analysis of expressed genes encoding cell cycle regulators and ribosomal protein subunits. **a** Expression clustering of cell cycle related genes in 0 DPA epidermal (left), 2 DPA epidermal (middle), 2 DPA fiber (right) of GhTM-1, Gb3–79, and GhMD17. Upregulation (yellow box) and down-regulation (green box) of genes in early fiber cells are indicated. **b** Expression clustering of putative ribosome protein subunit genes in 0 DPA epidermal (left), 2 DPA epidermal (middle), 2 DPA fiber (right) of GhTM-1, Gb3–79, and GhMD17. Upregulation (yellow box) and down-regulation (green box) in early fiber cells are indicated. **Supplemental Figure 6.** Analysis of fiber cell numbers in cultured ovules. **a** Number of fiber cells per 1 mm^2^ on ovule surface of GhTM-1 incubated in culture for 2 days with control (grey), Rbin-1 (blue), and Roscovitine (red) treatments. Two asterisks indicate a statistical significance level of *P* < 0.01 (Student’s *t* test). **b** Photos of epidermis showing fiber cell initials in GhTM-1 incubated with mock (control, top), Rbin-1 (middle), and Roscovitine (bottom). Bars = 60 μm. **Supplemental Figure 7.** Phylogenetic analysis of upregulated and down-regulated *ARFs* in cotton fibers and their *Arabidopsis* homologs. Phylogenetic tree of all *ARF* genes in cotton and *Arabidopsis*. Gene names in the figure were identified in the list of upregulated and down-regulated genes in cotton fibers and their homologs in *Arabidopsis*. Color of line in phylogenetic tress represents upregulated (red), down-regulated (blue) genes, both up and down-regulated genes (green, *ARF19*) in fibers. **Supplemental Figure 8.** Phylogenetic analysis of upregulated and down-regulated MYB transcription factor genes in cotton fibers and their *Arabidopsis* homologs. Phylogenetic trees of all MYB transcription factor genes in cotton and *Arabidopsis*. Gene names in the figure were identified in the list of upregulated and down-regulated genes in cotton early fiber cells and their homologs in *Arabidopsis*. Genes described and named in previous *Gossypium* studies listed in parenthesis. Color of line in phylogenetic tress represents up-regulated (red) and down-regulated (blue) genes in fibers, green are other genes known to be upregulated in fibers which were not detected in this experiment.

**Additional file 2: Supplemental Table 1.** RNA-seq statistics of LCM samples. **Supplemental Table 2.** Upregulated and down-regulated genes in fiber cell initials at 0 DPA. **Supplemental Table 3.** Upregulated and down-regulated genes in early fiber cells at 2 DPA. **Supplemental Table 3a.** Up-regulated and down-regulated genes in epidermal cells in 0 and 2 DPA. **Supplemental Table 4.** Upregulated genes in early fiber cells (0 and 2 DPA) specific to species. **Supplemental Table 4a.** Upregulated and down-regulated genes in fiber cells between GhTM-1 and Gb3–79 at 2 DPA. **Supplemental Table 4b.** Significant GO terms in upregulated and down-regulated genes between GhTM-1 and Gb3–79 in fiber cells at 2 DPA. **Supplemental Table 5.** List of A and D homoeologs with biased expression values. **Supplemental Table 5a**. Orthologs of G. hirstum and *G. barbadense*. **Supplemental Table 6.** List of putative ribosomal protein subunit and cell cycle genes with expression values in LCM samples. **Supplemental Table 7.** Expression values of putative *TOR*, *RAPTOR*, *LST8*, and *EPB1* genes in LCM samples*.* **Supplemental Table 8.** Expression values of phytohormone and MYB transcription factor related genes in LCM samples.

## Data Availability

The mRNA sequencing data have been deposited in the NCBI Gene Expression Omnibus (GEO) database, https://www.ncbi.nlm.nih.gov/geo (accession GSE152995).

## Abbreviations

LCM: Laser-capture microdissection
MYB: Myeloblastosis
DAP: Days post anthesis
TOR: TARGET OF RAPAMYCIN
FPKM: Fragments per kilobase per million
PCA: Principal component analysis
EBP1: ErbB-3 EPIDERMAL GROWTH FACTOR BINDING PROTEIN1
LST8: LETHAL WITH SEC THIRTEEN PROTEIN8
