# Supplementary material for: LCM and RNA-seq analyses revealed roles of cell cycle and translational regulation and homoeolog expression bias in cotton fiber cell initiation
Source: BMC Genomics. 2021 Apr 29;22:309. doi: 10.1186/s12864-021-07579-1 (PMC8082777; doi:10.1186/s12864-021-07579-1)
Supplement: Supplementary file 1 — Additional file 1: Supplemental Figure 1. Images of Laser Capture Microdissection (LCM) and RNA quality in LCM samples. a Isolation of fiber and epidermal cell tissues by Laser Capture Microdissection (LCM) method. Top represents epidermal cell isolation from ovules at 0 DPA in GhTM-1 and Gb3–79, and GhMD17. Bottom shows separation of fiber and epidermal cell layers from ovules at 2 DPA in GhTM-1 and Gb3–79. Bar represents 150 μm. b Line plots showing RNA size (x-axis) and quantity (y-axis) measured by Bioanalyzer with RIN number in GhTM-1 epidermal cells (0 DPA) (left) and GhTM-1 fiber cells (2 DPA) (right). Two ribosomal RNA peaks are clearly visible. Supplemental Figure 2. Gene expression correlation between GhTM-1, Gb3–79, and GhMD17 and down-regulated genes in early fiber cells. a Pearson’s correlation coefficients of LCM RNA-seq samples averaged between 2 replicates. Hierarchical clustering was based on geometric distance. b Venn diagram analysis of down-regulated genes in early fiber cells at 0 DPA; GhMD17 > GhTM-1 (blue) and GhMD17 > Gb3–79 (red). Shared down-regulated genes in fibers at 0 DPA (white) between comparisons are shown in dashed box. c Venn diagram analysis of down-regulated genes in early fiber cells at 2 DPA; GhTM-1(epi > fiber) (blue), Gh3–79 (epi > fiber) (red), GhMD17(epi) > GhTM-1(fiber) (yellow), and GhMD17(epi) > Gb3–79(fiber) (green). Shared down-regulated genes in early fiber cells at 2 DPA (white) between comparisons are shown in dashed box. Supplemental Figure 3. Gene expression divergence between GhTM-1 and Gb3–79 in epidermal cells at 0 and 2 DPA. a Venn Diagram analysis of upregulated genes in epidermal cells at 0 DPA; GhTM-1 0epi > 2epi (light blue) and Gb3–79 0epi > 2epi (pink). b Venn Diagram analysis of upregulated genes in epidermal cells at 2 DPA; GhTM-1 2epi > 0epi (blue) and Gb3–79 2epi > 0epi (red). Supplemental Figure 4. Biased expression of A and D homoeologs in GhTM-1 and Gb3–79. a Number of A and D homoeologs (pairs), [file 12864_2021_7579_MOESM1_ESM.pptx]

## Slide 1
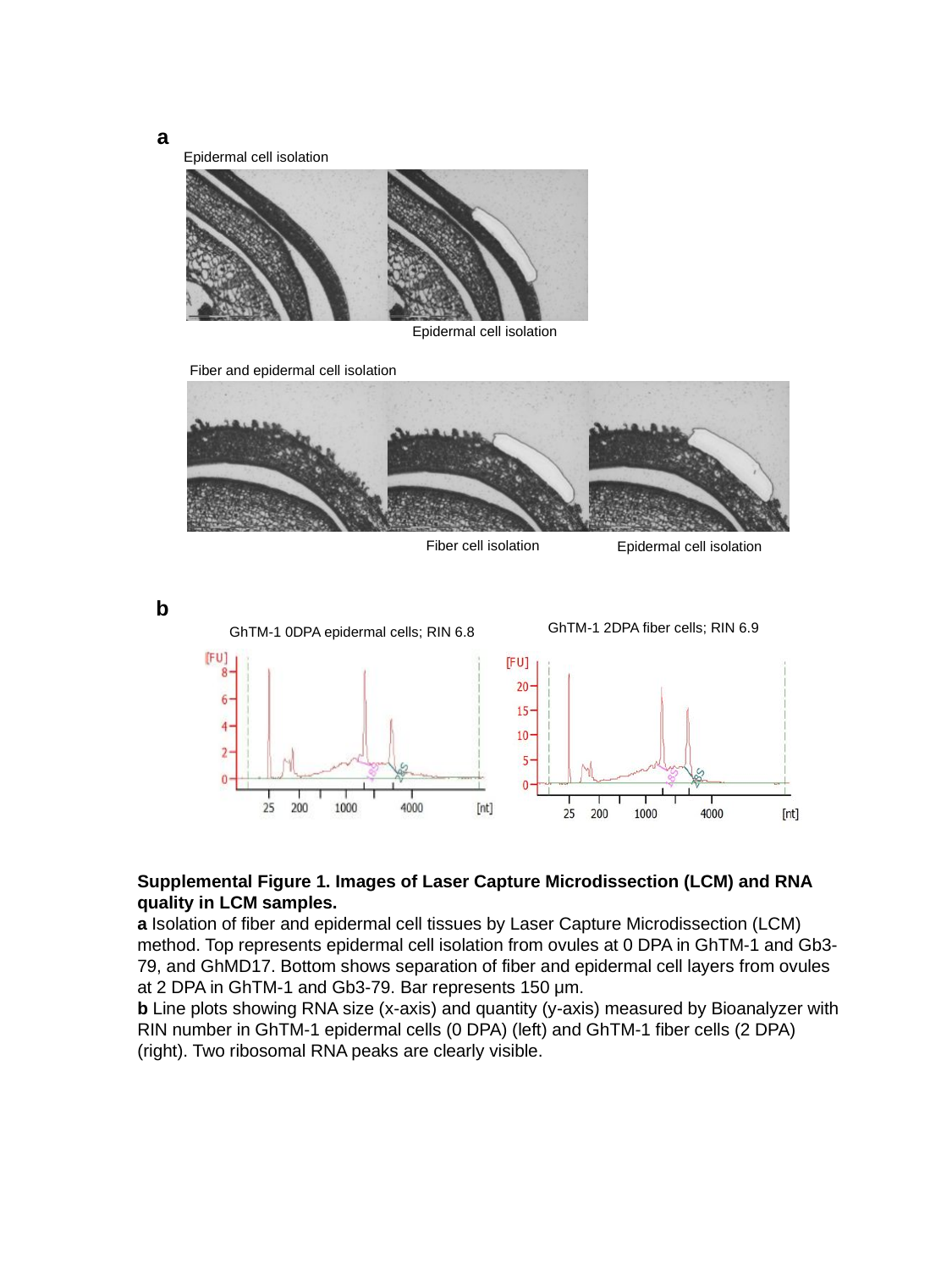

a
Epidermal cell isolation
Epidermal cell isolation
Fiber and epidermal cell isolation
Fiber cell isolation
Epidermal cell isolation
b
GhTM-1 2DPA fiber cells; RIN 6.9
GhTM-1 0DPA epidermal cells; RIN 6.8
Supplemental Figure 1. Images of Laser Capture Microdissection (LCM) and RNA quality in LCM samples.
a Isolation of fiber and epidermal cell tissues by Laser Capture Microdissection (LCM) method. Top represents epidermal cell isolation from ovules at 0 DPA in GhTM-1 and Gb3-79, and GhMD17. Bottom shows separation of fiber and epidermal cell layers from ovules at 2 DPA in GhTM-1 and Gb3-79. Bar represents 150 μm.
b Line plots showing RNA size (x-axis) and quantity (y-axis) measured by Bioanalyzer with RIN number in GhTM-1 epidermal cells (0 DPA) (left) and GhTM-1 fiber cells (2 DPA) (right). Two ribosomal RNA peaks are clearly visible.

## Slide 2
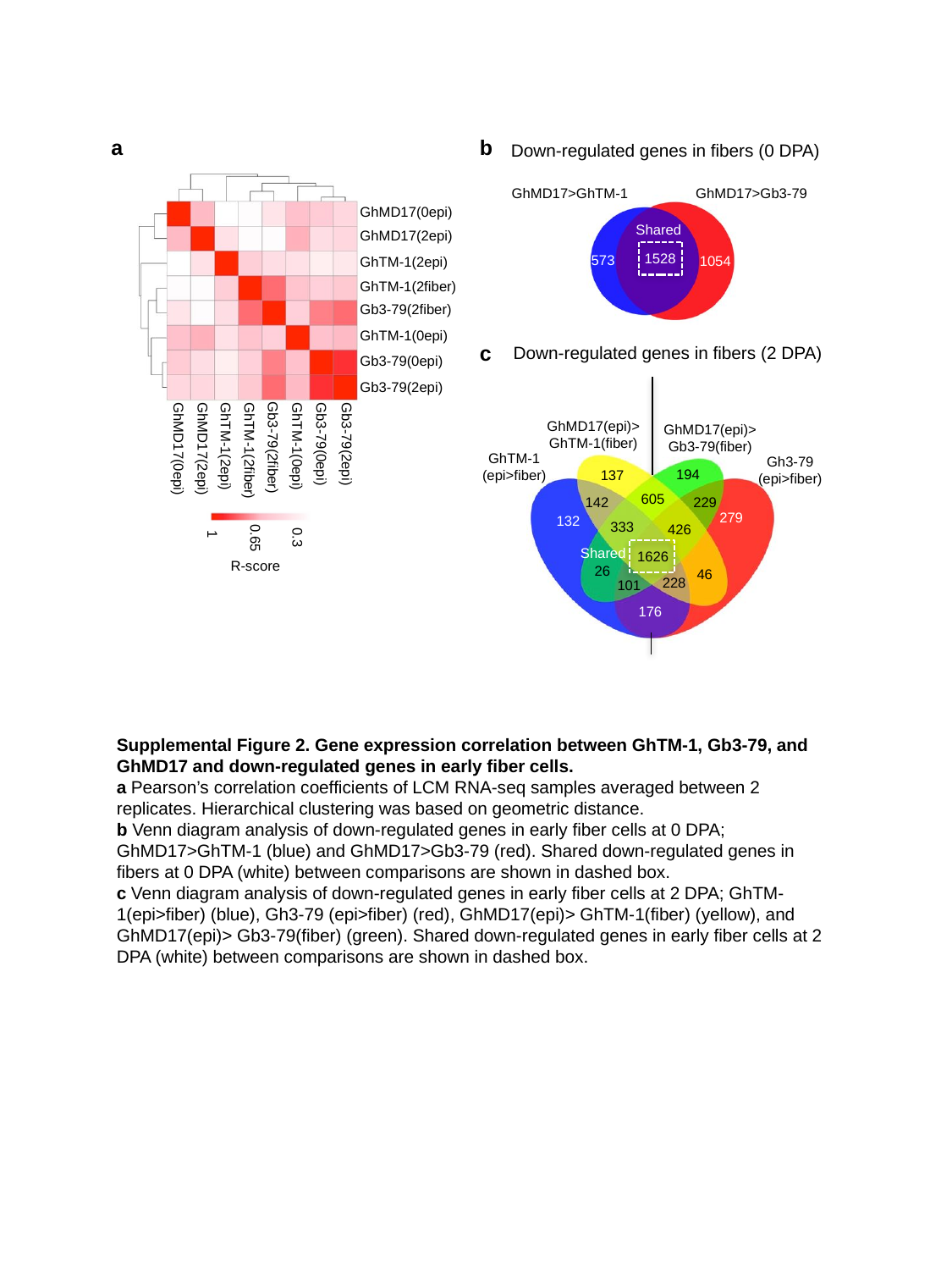

a
b
Down-regulated genes in fibers (0 DPA)
GhMD17>GhTM-1
GhMD17>Gb3-79
GhMD17(0epi)
Shared
GhMD17(2epi)
1528
573
1054
GhTM-1(2epi)
GhTM-1(2fiber)
Gb3-79(2fiber)
GhTM-1(0epi)
c
Down-regulated genes in fibers (2 DPA)
Gb3-79(0epi)
Gb3-79(2epi)
GhMD17(epi)>
GhTM-1(fiber)
GhMD17(epi)>
Gb3-79(fiber)
Gb3-79(0epi)
Gb3-79(2epi)
GhTM-1(2epi)
GhTM-1(0epi)
Gb3-79(2fiber)
GhMD17(0epi)
GhMD17(2epi)
GhTM-1(2fiber)
GhTM-1 (epi>fiber)
Gh3-79 (epi>fiber)
194
137
605
229
142
279
132
333
426
1
0.3
0.65
Shared
1626
R-score
26
46
228
101
176
Supplemental Figure 2. Gene expression correlation between GhTM-1, Gb3-79, and GhMD17 and down-regulated genes in early fiber cells.
a Pearson’s correlation coefficients of LCM RNA-seq samples averaged between 2 replicates. Hierarchical clustering was based on geometric distance.
b Venn diagram analysis of down-regulated genes in early fiber cells at 0 DPA; GhMD17>GhTM-1 (blue) and GhMD17>Gb3-79 (red). Shared down-regulated genes in fibers at 0 DPA (white) between comparisons are shown in dashed box.
c Venn diagram analysis of down-regulated genes in early fiber cells at 2 DPA; GhTM-1(epi>fiber) (blue), Gh3-79 (epi>fiber) (red), GhMD17(epi)> GhTM-1(fiber) (yellow), and GhMD17(epi)> Gb3-79(fiber) (green). Shared down-regulated genes in early fiber cells at 2 DPA (white) between comparisons are shown in dashed box.

## Slide 3
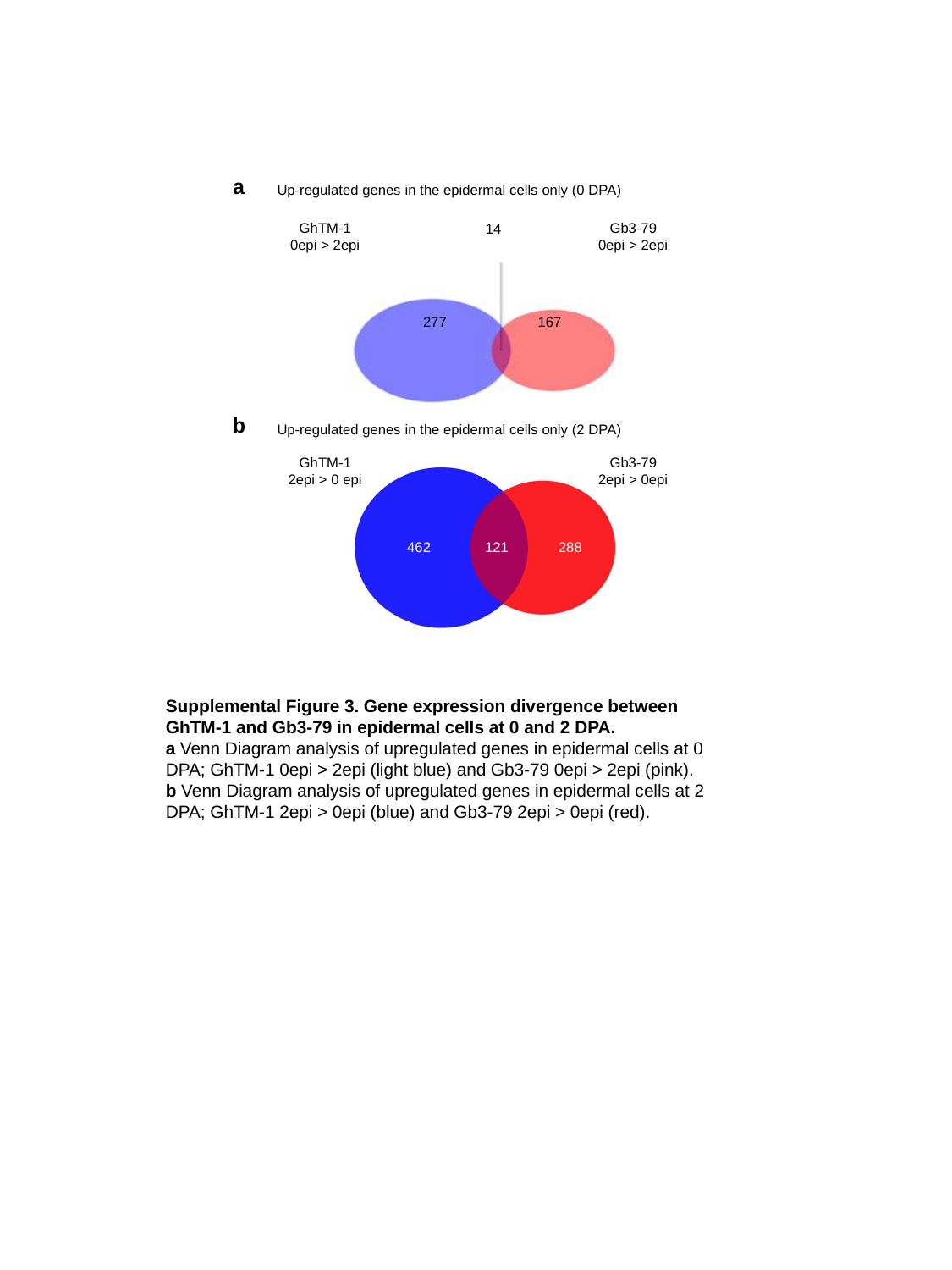

a
Up-regulated genes in the epidermal cells only (0 DPA)
GhTM-1
0epi > 2epi
Gb3-79
0epi > 2epi
14
277
167
b
Up-regulated genes in the epidermal cells only (2 DPA)
GhTM-1
2epi > 0 epi
Gb3-79
2epi > 0epi
462
121
288
Supplemental Figure 3. Gene expression divergence between GhTM-1 and Gb3-79 in epidermal cells at 0 and 2 DPA.
a Venn Diagram analysis of upregulated genes in epidermal cells at 0 DPA; GhTM-1 0epi > 2epi (light blue) and Gb3-79 0epi > 2epi (pink).
b Venn Diagram analysis of upregulated genes in epidermal cells at 2 DPA; GhTM-1 2epi > 0epi (blue) and Gb3-79 2epi > 0epi (red).

## Slide 4
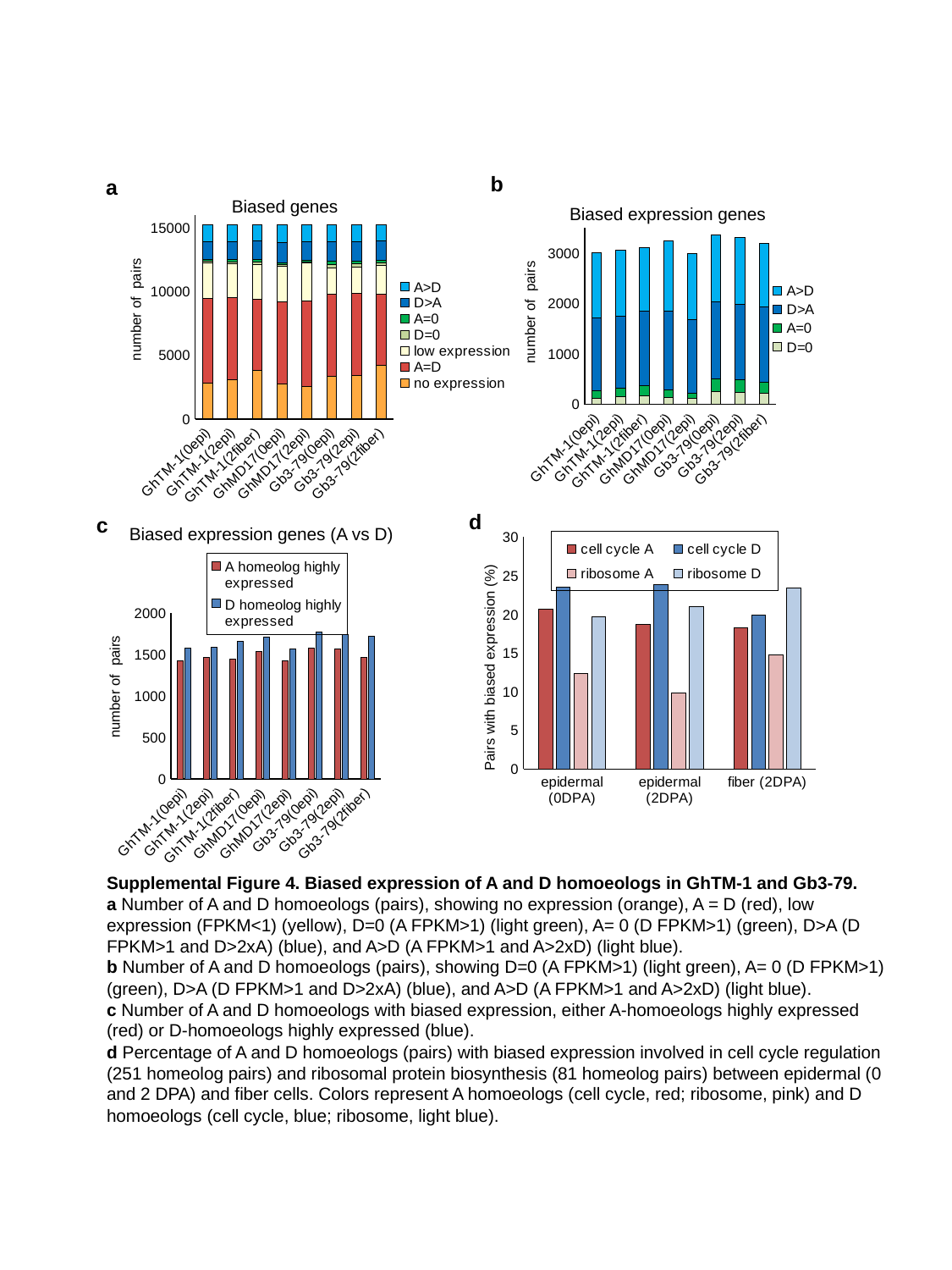

b
a
Biased genes
Biased expression genes
### Chart
| Category | no expression | A=D | low expression | D=0 | A=0 | D>A | A>D |
|---|---|---|---|---|---|---|---|
| GhTM-1(0epi) | 2843.0 | 6595.0 | 2779.0 | 128.0 | 138.0 | 1441.0 | 1296.0 |
| GhTM-1(2epi) | 3046.0 | 6502.0 | 2616.0 | 152.0 | 166.0 | 1424.0 | 1314.0 |
| GhTM-1(2fiber) | 3801.0 | 5593.0 | 2716.0 | 175.0 | 195.0 | 1471.0 | 1269.0 |
| GhMD17(0epi) | 2771.0 | 6442.0 | 2763.0 | 133.0 | 159.0 | 1550.0 | 1402.0 |
| GhMD17(2epi) | 2532.0 | 6747.0 | 2947.0 | 112.0 | 115.0 | 1450.0 | 1317.0 |
| Gb3-79(0epi) | 3346.0 | 6447.0 | 2069.0 | 259.0 | 255.0 | 1521.0 | 1323.0 |
| Gb3-79(2epi) | 3385.0 | 6495.0 | 2031.0 | 236.0 | 252.0 | 1492.0 | 1329.0 |
| Gb3-79(2fiber) | 4227.0 | 5574.0 | 2229.0 | 219.0 | 224.0 | 1497.0 | 1250.0 |
### Chart
| Category | D=0 | A=0 | D>A | A>D |
|---|---|---|---|---|
| GhTM-1(0epi) | 128.0 | 138.0 | 1441.0 | 1296.0 |
| GhTM-1(2epi) | 152.0 | 166.0 | 1424.0 | 1314.0 |
| GhTM-1(2fiber) | 175.0 | 195.0 | 1471.0 | 1269.0 |
| GhMD17(0epi) | 133.0 | 159.0 | 1550.0 | 1402.0 |
| GhMD17(2epi) | 112.0 | 115.0 | 1450.0 | 1317.0 |
| Gb3-79(0epi) | 259.0 | 255.0 | 1521.0 | 1323.0 |
| Gb3-79(2epi) | 236.0 | 252.0 | 1492.0 | 1329.0 |
| Gb3-79(2fiber) | 219.0 | 224.0 | 1497.0 | 1250.0 |number of pairs
number of pairs
### Chart
| Category | cell cycle A | cell cycle D | ribosome A | ribosome D |
|---|---|---|---|---|
| epidermal (0DPA) | 20.717131474103585 | 23.50597609561753 | 12.345679012345679 | 19.753086419753085 |
| epidermal (2DPA) | 18.725099601593627 | 23.904382470119522 | 9.876543209876543 | 20.98765432098765 |
| fiber (2DPA) | 18.326693227091635 | 19.9203187250996 | 14.814814814814813 | 23.456790123456788 |d
c
Biased expression genes (A vs D)
### Chart
| Category | A homeolog highly expressed | D homeolog highly expressed |
|---|---|---|
| GhTM-1(0epi) | 1424.0 | 1579.0 |
| GhTM-1(2epi) | 1466.0 | 1590.0 |
| GhTM-1(2fiber) | 1444.0 | 1666.0 |
| GhMD17(0epi) | 1535.0 | 1709.0 |
| GhMD17(2epi) | 1429.0 | 1565.0 |
| Gb3-79(0epi) | 1582.0 | 1776.0 |
| Gb3-79(2epi) | 1565.0 | 1744.0 |
| Gb3-79(2fiber) | 1469.0 | 1721.0 |Pairs with biased expression (%)
number of pairs
Supplemental Figure 4. Biased expression of A and D homoeologs in GhTM-1 and Gb3-79.
a Number of A and D homoeologs (pairs), showing no expression (orange), A = D (red), low expression (FPKM<1) (yellow), D=0 (A FPKM>1) (light green), A= 0 (D FPKM>1) (green), D>A (D FPKM>1 and D>2xA) (blue), and A>D (A FPKM>1 and A>2xD) (light blue).
b Number of A and D homoeologs (pairs), showing D=0 (A FPKM>1) (light green), A= 0 (D FPKM>1) (green), D>A (D FPKM>1 and D>2xA) (blue), and A>D (A FPKM>1 and A>2xD) (light blue).
c Number of A and D homoeologs with biased expression, either A-homoeologs highly expressed (red) or D-homoeologs highly expressed (blue).
d Percentage of A and D homoeologs (pairs) with biased expression involved in cell cycle regulation (251 homeolog pairs) and ribosomal protein biosynthesis (81 homeolog pairs) between epidermal (0 and 2 DPA) and fiber cells. Colors represent A homoeologs (cell cycle, red; ribosome, pink) and D homoeologs (cell cycle, blue; ribosome, light blue).

## Slide 5
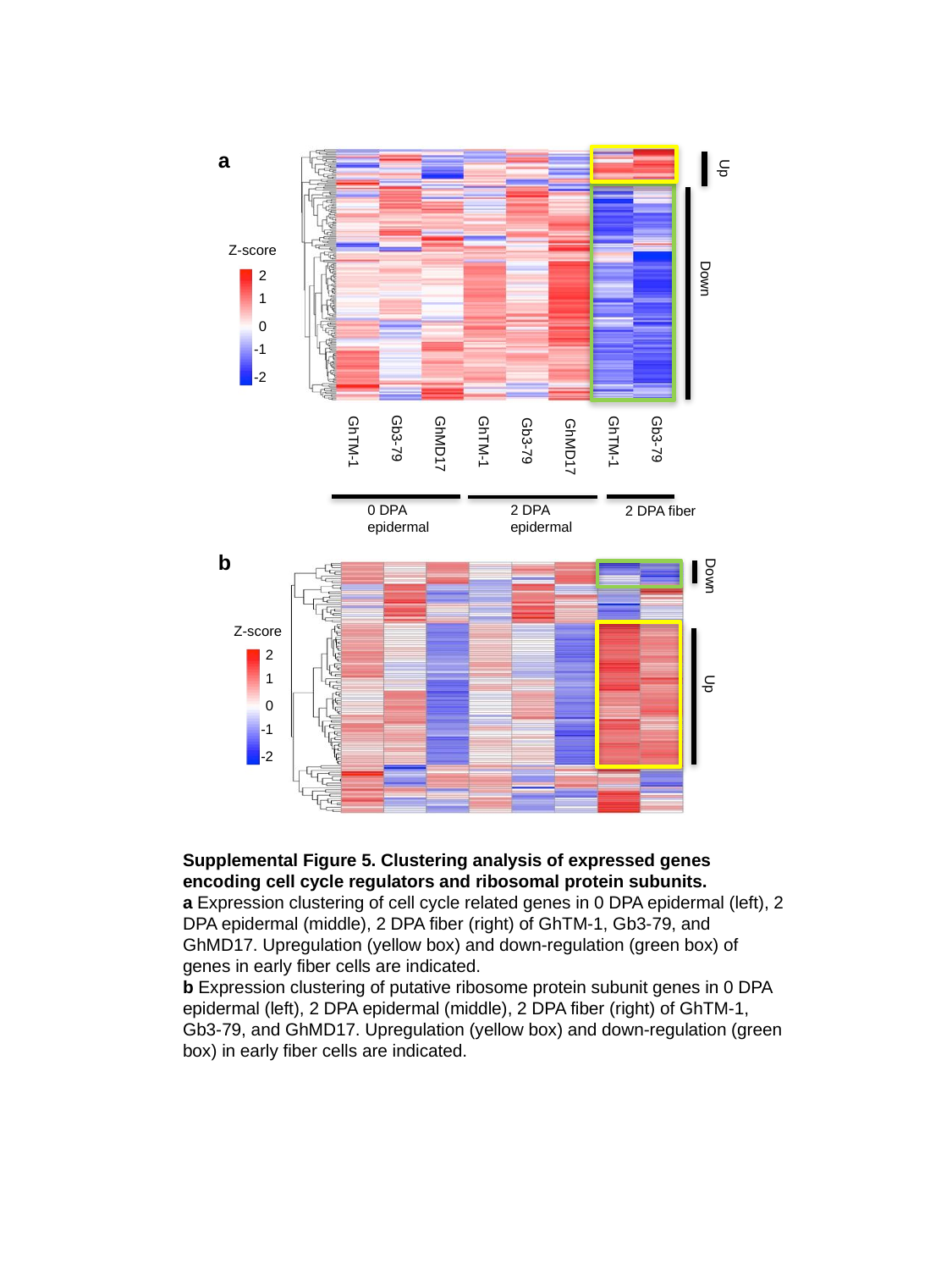

a
Up
Cell cycle regulation genes (231 genes)
Z-score
2
0
-2
Down
1
-1
Gb3-79
Gb3-79
Gb3-79
GhTM-1
GhTM-1
GhTM-1
GhMD17
GhMD17
2 DPA
epidermal
0 DPA
epidermal
2 DPA fiber
b
Down
Ribosomal proteins subunit genes (121 proteins)
Z-score
2
0
-2
1
-1
Up
Supplemental Figure 5. Clustering analysis of expressed genes encoding cell cycle regulators and ribosomal protein subunits.
a Expression clustering of cell cycle related genes in 0 DPA epidermal (left), 2 DPA epidermal (middle), 2 DPA fiber (right) of GhTM-1, Gb3-79, and GhMD17. Upregulation (yellow box) and down-regulation (green box) of genes in early fiber cells are indicated.
b Expression clustering of putative ribosome protein subunit genes in 0 DPA epidermal (left), 2 DPA epidermal (middle), 2 DPA fiber (right) of GhTM-1, Gb3-79, and GhMD17. Upregulation (yellow box) and down-regulation (green box) in early fiber cells are indicated.

## Slide 6
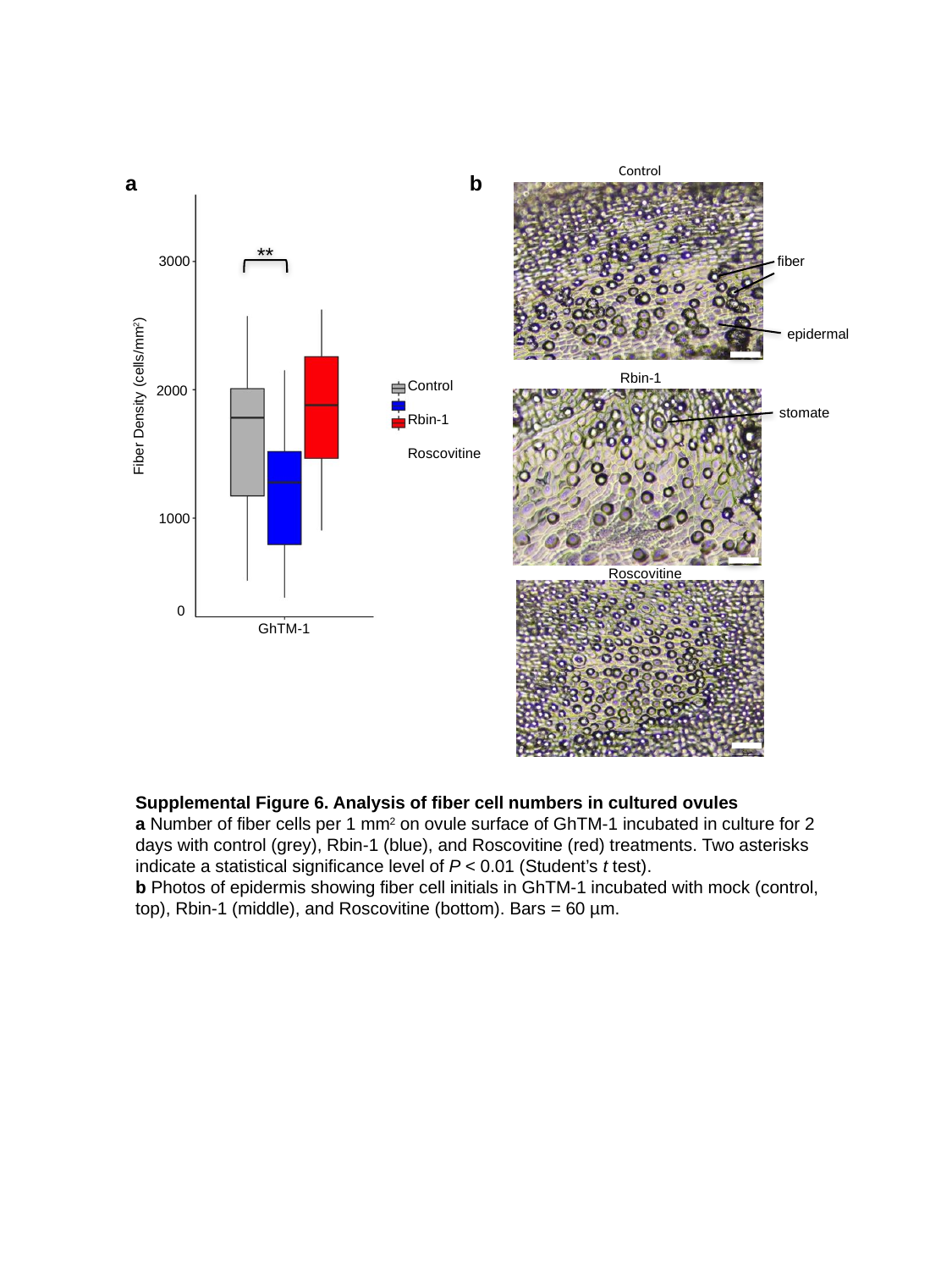

Control
a
b
**
3000
fiber
epidermal
Rbin-1
Control
Rbin-1
Roscovitine
2000
 Fiber Density (cells/mm2)
stomate
1000
Roscovitine
0
GhTM-1
Supplemental Figure 6. Analysis of fiber cell numbers in cultured ovules
a Number of fiber cells per 1 mm2 on ovule surface of GhTM-1 incubated in culture for 2 days with control (grey), Rbin-1 (blue), and Roscovitine (red) treatments. Two asterisks indicate a statistical significance level of P < 0.01 (Student’s t test).
b Photos of epidermis showing fiber cell initials in GhTM-1 incubated with mock (control, top), Rbin-1 (middle), and Roscovitine (bottom). Bars = 60 µm.

## Slide 7
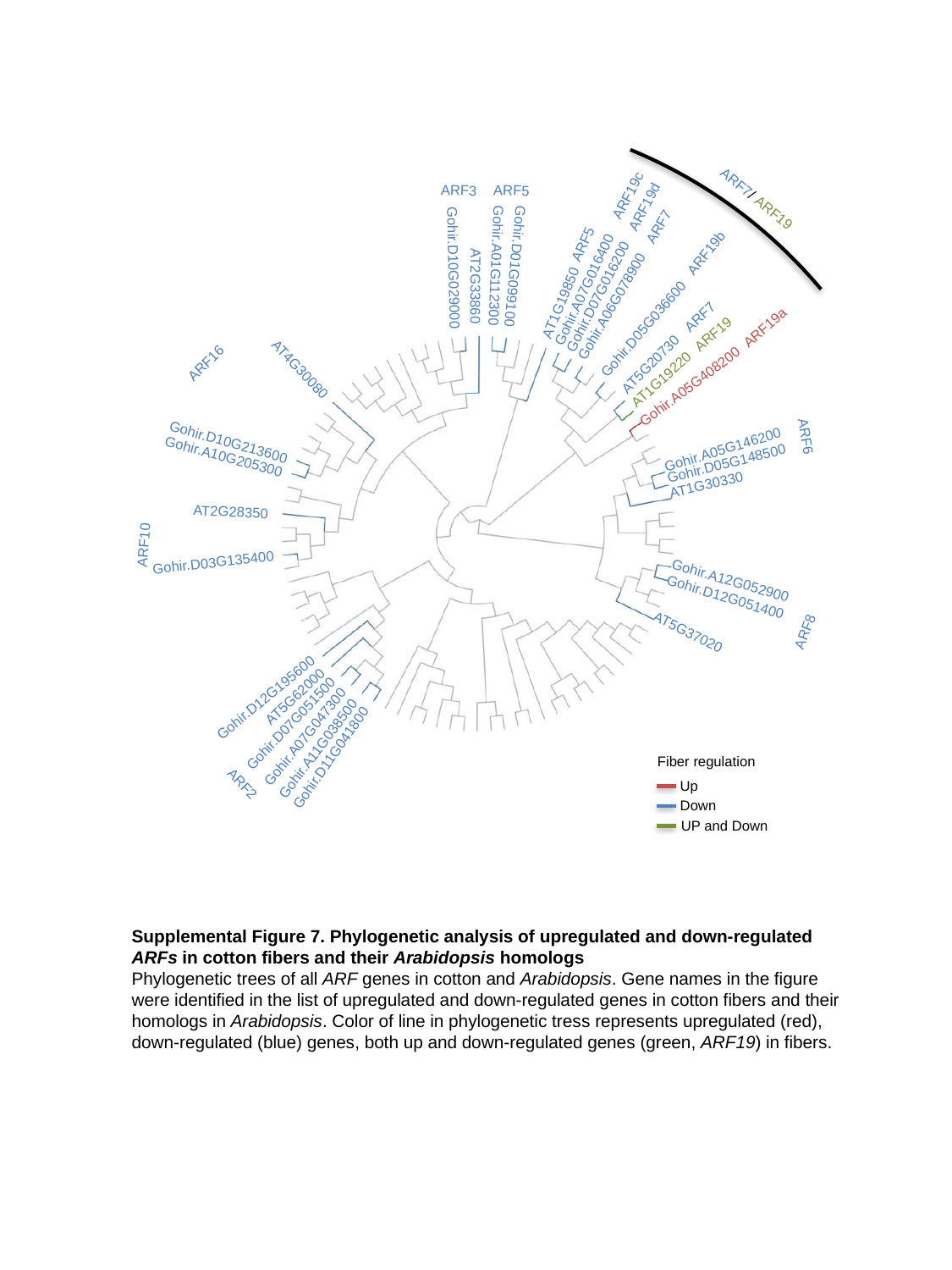

ARF3
ARF5
ARF19c
ARF7
ARF5
Gohir.A01G112300
Gohir.D01G099100
Gohir.D10G029000
AT2G33860
Gohir.A07G016400
Gohir.D07G016200
AT1G19850
Gohir.A06G078900
ARF7
Gohir.D05G036600
ARF19
ARF16
AT5G20730
AT4G30080
AT1G19220
Gohir.A05G408200
ARF6
Gohir.D10G213600
Gohir.A05G146200
Gohir.A10G205300
Gohir.D05G148500
AT1G30330
AT2G28350
ARF10
Gohir.D03G135400
Gohir.A12G052900
Gohir.D12G051400
AT5G37020
ARF8
AT5G62000
Gohir.D12G195600
Gohir.D07G051500
Gohir.A07G047300
Gohir.A11G038500
Gohir.D11G041800
Fiber regulation
ARF2
Up
Down
UP and Down
ARF7/ ARF19
ARF19d
ARF19b
ARF19a
Supplemental Figure 7. Phylogenetic analysis of upregulated and down-regulated ARFs in cotton fibers and their Arabidopsis homologs
Phylogenetic trees of all ARF genes in cotton and Arabidopsis. Gene names in the figure were identified in the list of upregulated and down-regulated genes in cotton fibers and their homologs in Arabidopsis. Color of line in phylogenetic tress represents upregulated (red), down-regulated (blue) genes, both up and down-regulated genes (green, ARF19) in fibers.

## Slide 8
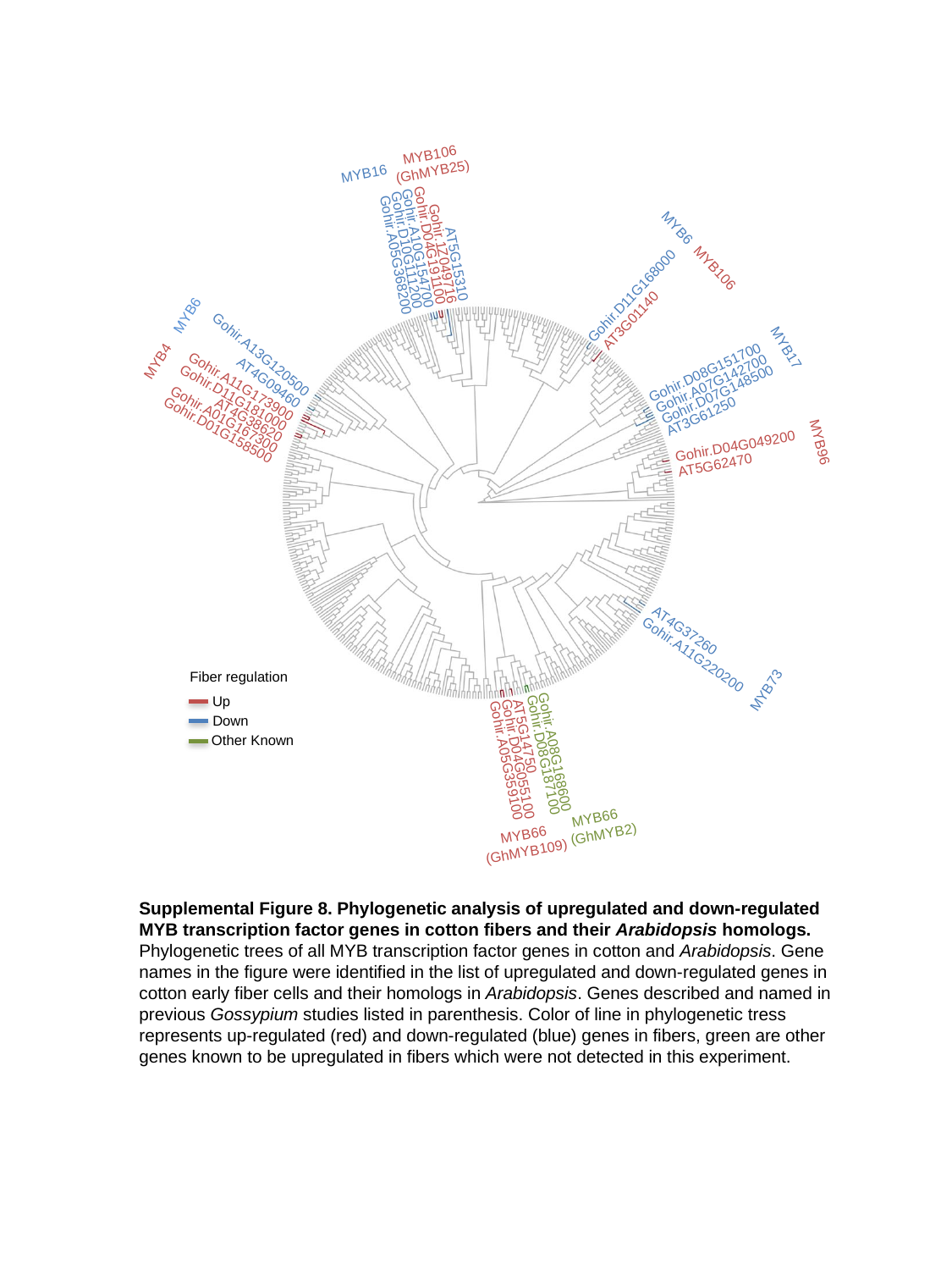

MYB106
(GhMYB25)
MYB16
MYB6 MYB106
Gohir.D04G191100
Gohir.A10G154700
Gohir.D10G111200
Gohir.A05G368200
Gohir.1Z049716
Gohir.D11G168000
AT5G15310
AT3G01140
MYB4 MYB6
MYB17
Gohir.A13G120500
Gohir.D08G151700
Gohir.A07G142700
Gohir.A11G173900
Gohir.D07G148500
AT4G09460
Gohir.D11G181000
AT3G61250
Gohir.A01G167300
Gohir.D01G158500
AT4G38620
MYB96
Gohir.D04G049200
AT5G62470
AT4G37260
Gohir.A11G220200
Fiber regulation
MYB73
Up
Down
Other Known
Gohir.A08G168600
Gohir.D08G187100
AT5G14750
Gohir.D04G055100
Gohir.A05G359100
MYB66
(GhMYB2)
MYB66
(GhMYB109)
Supplemental Figure 8. Phylogenetic analysis of upregulated and down-regulated MYB transcription factor genes in cotton fibers and their Arabidopsis homologs.
Phylogenetic trees of all MYB transcription factor genes in cotton and Arabidopsis. Gene names in the figure were identified in the list of upregulated and down-regulated genes in cotton early fiber cells and their homologs in Arabidopsis. Genes described and named in previous Gossypium studies listed in parenthesis. Color of line in phylogenetic tress represents up-regulated (red) and down-regulated (blue) genes in fibers, green are other genes known to be upregulated in fibers which were not detected in this experiment.
